# Proton Pump Inhibitor Use Exceeding the U.S. Food and Drug Administration Approved Treatment Duration for Patients With Peptic Ulcer Disease: A Retrospective Cohort Study

**DOI:** 10.1002/pds.70152

**Published:** 2025-04-29

**Authors:** Jordan A. Villars, Timothy S. Anderson, Jonathan G. Yabes, Robert E. Schoen, Ravy K. Vajravelu

**Affiliations:** ^1^ Department of Medicine University of Pittsburgh School of Medicine Pittsburgh Pennsylvania USA; ^2^ Division of General Internal Medicine, Department of Medicine University of Pittsburgh School of Medicine Pittsburgh Pennsylvania USA; ^3^ CHERP VA Pittsburgh Healthcare System Pittsburgh Pennsylvania USA; ^4^ Division of Gastroenterology, Hepatology and Nutrition, Department of Medicine University of Pittsburgh School of Medicine Pittsburgh Pennsylvania USA

**Keywords:** deprescribing, overuse, proton pump inhibitors

## Abstract

**Background:**

Proton‐pump inhibitors (PPIs) are effective in treating peptic ulcer disease (PUD), but they are often prescribed beyond the approved duration. Because PPIs are associated with adverse effects, there is a need for effective stewardship.

**Objective:**

To identify the frequency of and healthcare factors associated with PPI prescriptions exceeding the approved eight‐week treatment duration for PUD.

**Methods:**

We conducted a retrospective cohort study of patients diagnosed with acute PUD without other indications for PPI use using data from the Veterans Health Administration in the United States. Exposures were patient, provider, and facility factors that could influence PPI prescribing. The outcome was time to a filled PPI prescription exceeding the approved treatment duration for PUD. Associations were assessed using a multivariable time‐to‐recurrent‐event model to calculate adjusted hazard ratios (aHR) and population‐attributable fractions. Patients who developed indications for long‐term PPI use were censored.

**Results:**

We identified 7708 patients with PUD who met eligibility criteria and received PUD treatment (median age 79 [IQR 71–85], 7% female). Thirty‐five percent had PPI prescriptions exceeding the approved duration for a median of 346 days (IQR 165–643) of overuse. On the patient level, inpatient PUD diagnosis (aHR 1.32, 95% CI 1.25–1.39), use of nonsteroidal anti‐inflammatory drugs (NSAIDs) (aHR 1.26, 95% CI 1.18–1.34), use of anticoagulants (aHR 1.25, 95% CI 1.13–1.38), and moderate frailty (1.15, 95% CI 1.06–1.26) had the strongest associations with filled PPI prescriptions exceeding the approved duration. On the health‐system level, inpatient PUD diagnosis had the highest peak population attributable fraction at 0.26, followed by NSAIDs and anticoagulants at 0.18.

**Conclusions:**

Markers of patient complexity and medication use not meeting gastroprotection guidelines are associated with inappropriate PPI persistence among patients with PUD. These data may inform future targeted PPI deprescribing programs.

**Abbreviations**
aHR: adjusted hazard ratio
CDW: Corporate Data Warehouse
FDA: U.S. Food and Drug Administration
ICD: International Classification of Diseases
NSAID: nonsteroidal anti‐inflammatory drug
PPI: proton pump inhibitor
PUD: peptic ulcer disease
VHA: Veterans Health Administration
VISN: Veterans Integrated Services Network

**Summary**

- Healthcare factors associated with the use of proton pump inhibitors (PPI) for the treatment of peptic ulcer disease (PUD) beyond the approved duration of 8 weeks are unknown.
- We conducted a retrospective cohort study of patients treated for PUD by the Veterans Health Administration in the United States from 1999 to 2022.
- Thirty‐five percent of patients treated for PUD had PPI exposure exceeding the approved treatment duration.
- Markers of patient complexity, such as being diagnosed with PUD as an inpatient and NSAID or anticoagulant use not meeting gastroprotection criteria, were associated with PPI prescriptions exceeding the approved treatment duration.
- These factors may inform the development of targeted deprescribing programs that reduce unindicated long‐term PPI use.

## 1 Introduction

Ten percent of Americans use proton pump inhibitors (PPIs), making them one of the most commonly used medications in the United States [1]. However, epidemiologic studies demonstrate that 60% of patients using PPIs do not have definitive indications for their use [2, 3, 4, 5, 6, 7, 8]. Furthermore, observational studies have raised concerns about the long‐term safety of PPIs [9, 10, 11], and a recent randomized controlled trial of pantoprazole versus placebo and its *post hoc* open‐label extension demonstrated that PPIs are associated with enteric infections and estimated glomerular filtration rate decline [12, 13]. These findings underscore the importance of medication stewardship to improve population‐level drug safety [6, 14, 15, 16].

Traditionally, stewardship of chronically used medications has focused on opportunistic deprescribing, in which providers identify and discontinue unindicated prescriptions during routine medical encounters [17]. Unfortunately, due to high provider workloads and patient complexity, opportunistic deprescribing is difficult to scale across health systems and could contribute to health inequities [18, 19]. An alternative approach is to apply deprescribing rules broadly across all patients. However, these programs are often nonspecific and result in patients with strong indications for therapy experiencing inappropriate deprescribing. For example, a recent study examining the effect of a region‐wide program to discontinue long‐term PPI prescriptions in the Veterans Health Administration (VHA) demonstrated a reduction in population‐level PPI use, but it also identified that patients at high risk for upper gastrointestinal bleeding had their gastroprotective PPIs deprescribed inappropriately [20]. Targeted deprescribing strategies based on healthcare factors associated with inappropriate chronic prescriptions are needed to balance the strengths and weaknesses of opportunistic and broad deprescribing programs.

To date, several methodologic barriers have prevented the use of real‐world health data to identify factors associated with inappropriate long‐term PPI use. These include difficulty ascertaining PPI indications, variable treatment duration recommendations, and high rates of over‐the‐counter PPI use that are not captured in health databases. Studying peptic ulcer disease (PUD) presents an opportunity to overcome these obstacles because it is a strong indication for PPI treatment for a maximum of 8 weeks based on clear regulatory and clinical guidance from the U.S. Food and Drug Administration (FDA) and professional societies [21, 22, 23, 24]. Additionally, utilizing VHA data provides the ability to reduce misclassification from over‐the‐counter PPI use because it is an integrated health system with strong prescription benefits. To leverage these analytic opportunities and guide the development of PPI deprescribing programs, we conducted a retrospective cohort study of VHA patients with PUD to understand healthcare factors associated with filled PPI prescriptions exceeding the approved treatment duration.

## 2 Methods

### 2.1 Data Source

We used data from 1999 to 2022 from the VHA Corporate Data Warehouse (CDW) to conduct this retrospective cohort study. The VHA is an integrated health system that provides primary and specialty medical care to Veterans of the United States Armed Services and a selection of their family members [25]. It consists of 172 medical centers and 1138 outpatient clinics that serve 9 million patients annually. CDW contains electronic health record and insurance claims data, including demographics, diagnosis codes, procedures, lab results, prescription information, and free‐text documents [26, 27].

### 2.2 Cohort Eligibility: Acute PUD Diagnosis

**FIGURE 1:**
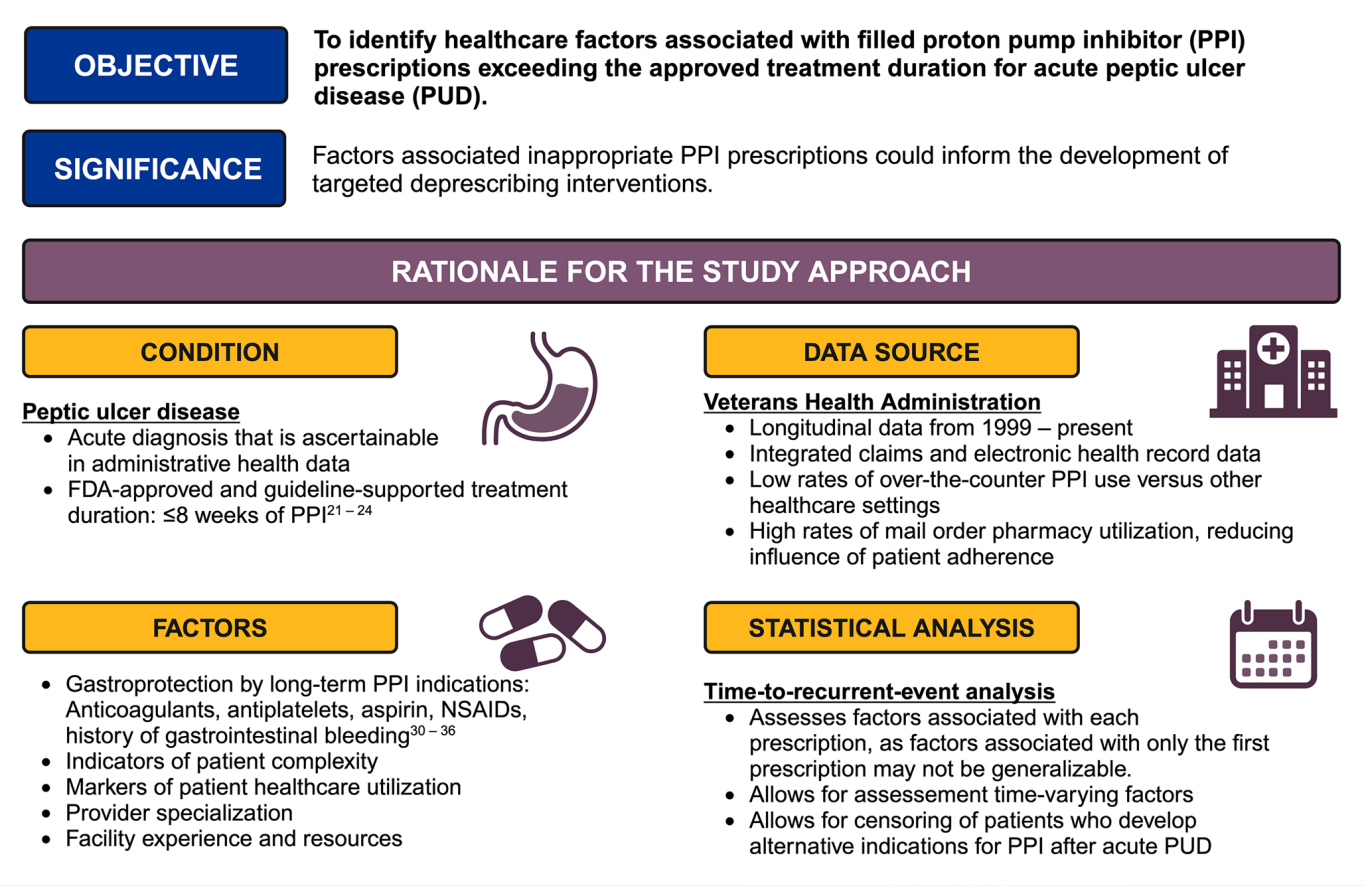
Study conceptual model and rationale.

Among patients meeting VHA enrollment criteria, we assembled a cohort of patients diagnosed with acute PUD. Potential patients were identified by International Classification of Diseases (ICD) codes for acute PUD from inpatient or outpatient encounters (Table S1). The date of the first acute PUD diagnosis was considered the cohort entry date. Because there is no validated case‐finding algorithm for acute PUD, we implemented several criteria to optimize the likelihood of identifying patients with clinically significant acute PUD. For inclusion, patients must have had an upper endoscopy in conjunction with their first acute PUD diagnosis and not have had prior diagnoses of chronic or unspecified PUD. Furthermore, because the outcome of interest was PPI prescriptions exceeding the approved treatment duration for PUD, we excluded patients with other indications for acute or chronic PPI use, such as Barrett's esophagus, and patients who had filled a PPI prescription between 365 and 14 days before the first acute PUD diagnosis. Finally, we identified a subset of patients who filled a prescription for a PPI in the VHA within 14 days of PUD diagnosis. Patients who did not fill a PPI prescription in the VHA within 14 days were not analyzed because they may obtain their prescriptions using non‐VHA benefits or they may have never initiated treatment [28, 29]. A full description and rationale of the cohort eligibility is described in Figure 1 and Data S1.

### 2.3 Outcome: Time to a Filled PPI Prescription Exceeding the Approved Treatment Duration

FDA‐approved prescribing instructions and PUD treatment guidelines recommend that PPI treatment last no longer than 8 weeks [21, 22, 23, 24]. As such, we classified filled PPI prescriptions that would extend treatment beyond 8 weeks to exceed the approved duration. To determine if a PPI prescription had a duration exceeding the approved length, we used 60 days as the threshold instead of 8 weeks × 7 days = 56 days because prescriptions in the United States are often written in 30‐day increments. A patient's backstock of PPI from previously filled prescriptions was not considered because providers do not routinely calculate remaining stock when making prescription decisions. Because active *H. pylori* infection is also an indication for up to 2 weeks of PPI and is causative of some cases of PUD, we accounted for *H. pylori* treatment by classifying filled prescriptions that ended beyond 60 days from the acute PUD diagnosis date but within 60 days of the first *H. pylori* diagnosis as having approved duration. The 60‐day margin after *H. pylori diagnosis* instead of a two‐week margin was implemented to allow for delays in treatment initiation after *H. pylori* diagnosis. *H. pylori* diagnosis was ascertained from a combination of ICD codes (ICD‐9041.86 and ICD‐10 B96.81), laboratory results (stool antigen, urea breath test, and *Campylobacter*‐like organism rapid urease test), and free‐text surgical pathology results. Recurrent and refractory *H. pylori* infections were not considered.

During the study period, several societies representing various medical specialties released highly variable recommendations for prophylaxis of gastrointestinal bleeding using PPIs. In a secondary analysis to assess the maximal effect of these guidelines on long‐term PPI prescribing, we considered PPI prescriptions among patients who met any of the gastroprotection guidelines as having an approved duration. Individual gastroprotection guidelines were assessed the year after their introduction and later. The gastroprotection criteria were history of PUD and NSAID use (after 2009) [30, 31, 32]; history of gastrointestinal bleeding (after 2010) [33]; antiplatelet use and any of age greater than 65, anticoagulant use, NSAID use, or aspirin use (after 2010) [33]; concurrent aspirin and antiplatelet use (after 2017) [34]; concurrent aspirin and anticoagulant use (after 2018) [35]; or two or more of aspirin, NSAIDs, anticoagulants, or antiplatelets (after 2020) [36].

### 2.4 Patient, Provider, and Facility Factors

Because the objective of the study was to identify healthcare factors associated with filled PPI prescriptions exceeding the approved treatment duration among patients with PUD, we assessed patient, provider, and facility factors that could influence prescribing and filling of PPIs. Patient factors included age at PUD diagnosis, sex, self‐reported race, self‐reported ethnicity, calendar year of PUD diagnosis, whether the PUD diagnosis was made during a hospitalization, whether the PUD diagnosis was associated with gastrointestinal bleeding, the Charlson‐Deyo comorbidity score, the VA Frailty Index, healthcare fragmentation through the usual provider of care index, and the VHA priority group at PUD diagnosis (an administrative categorization that influences VHA enrollment and the cost of copays based on a Veteran's military service, disability, income, and other benefits) [37, 38, 39, 40]. Provider factors included provider type (physician versus advanced practice provider), provider specialty, and provider subspecialty at the time of PPI prescription. Facility factors included Veterans Integrated Services Network (VISN, an organizational unit of regional healthcare delivery in the VHA) and rurality of the VHA facility where the patient's PUD diagnosis was made. A conceptual model of the relationship between these factors and the study outcome is presented in Figure S1.

As described in the *Outcome* subsection, we ascertained whether each patient in the cohort met criteria for several gastroprotection guidelines. To do so, we identified aspirin, antiplatelet, anticoagulant, or nonsteroidal anti‐inflammatory drug (NSAID) use at each opportunity to receive a PPI prescription (specific agents in Table S2). We also identified H2‐receptor antagonist use, as these may serve as substitutes for PPIs. Methodology for medication exposure ascertainment is described in Data S1.

### 2.5 Statistical Analysis

This analysis included three steps. First, we calculated the proportion of patients in the acute PUD cohort who filled PPI prescriptions exceeding the approved treatment duration and described the characteristics of these patients and prescriptions. We reported counts and percentages for categorical variables and medians and interquartile ranges (IQR) for continuous variables. Second, to assess which healthcare factors explain the receipt of a PPI prescription exceeding the approved treatment duration for PUD, we developed a multivariable Andersen‐Gill model using time‐to‐recurrent‐event data to calculate the adjusted hazard ratio (aHR) for each factor [41]. The acute PUD cohort entry date was the start of follow‐up and the fill dates of PPI prescriptions exceeding the approved duration were the outcomes. We chose the Andersen‐Gill model for recurrent events over the single‐event Cox Proportional Hazard model because patients can receive PPI prescriptions several times after an acute PUD diagnosis. Furthermore, limiting the analysis to only the first PPI prescription exceeding the approved treatment duration may not reflect factors associated with later prescriptions. Model building details are described in Data S1. Third, to understand the health system‐wide impact of a particular factor on filled PPI prescriptions exceeding the approved treatment duration, we calculated the adjusted time‐to‐event population attributable fraction (details in Data S1) [42]. We interpreted the adjusted population attributable fraction as the proportion of PPI prescriptions exceeding the approved duration that would be eliminated from the health system if patients with the factor were treated equivalently to patients without the factor.

Because the exclusion diagnoses described in the *Cohort Eligibility* subsection are absolute or relative indications for PPI, patients who experienced these after PUD diagnosis were censored from the analysis. Additionally, to analyze only healthcare delivery that is potentially influenced by the PUD diagnosis, all patients were censored after 3 years. Furthermore, because of a network‐wide PPI deprescribing initiative conducted in VISN 17 starting on August 1, 2013, patients who had their PUD diagnosis in VISN 17 were censored after this date [20]. In the secondary analysis, patients meeting gastroprotection criteria were censored at the time they qualified under any guideline. Figure S2 depicts the cohort eligibility, outcome, healthcare factor, and censoring assessment intervals.

Data extraction from VHA CDW was conducted using the R statistical programming environment (Vienna, Austria). Data analysis was conducted using Stata MP 18.0 (College Station, TX). Figure 1 and Figure S2 were designed in BioRender. This research was classified as exempt human subjects research by the VA Pittsburgh Healthcare System Institutional Review Board.

### 2.6 Sensitivity Analysis

We conducted four sensitivity analyses of the primary analysis to assess the impact of key study design elements. First, to determine the impact of limiting the study follow‐up to 3 years, we performed a sensitivity analysis in which the maximum follow‐up time was extended to 5 years. Second, to assess the maximal impact of not accounting for delayed gastric ulcer healing on repeat upper endoscopy in the main analysis, we performed a sensitivity analysis where PPIs prescribed in the 60 days after follow‐up upper endoscopies were to have approved treatment duration. Third, to reduce potential heterogeneity in provider decision‐making about concomitant antiplatelet and PPI prescriptions related to the 2009 U.S. Food and Drug Administration warning about drug–drug interactions, we conducted a sensitivity analysis limiting the cohort to patients diagnosed with PUD in 2010 and later [43]. Fourth, because there may be unmeasured confounding with indications for anticoagulants and antiplatelets that could influence the association of the VA Frailty Index with PPI prescriptions, we conducted a sensitivity analysis in which the VA Frailty Index was calculated without indications for anticoagulants or antiplatelets (atrial fibrillation, cerebrovascular disease, coronary artery disease, and peripheral vascular disease).

## 3 Results

**FIGURE 2:**
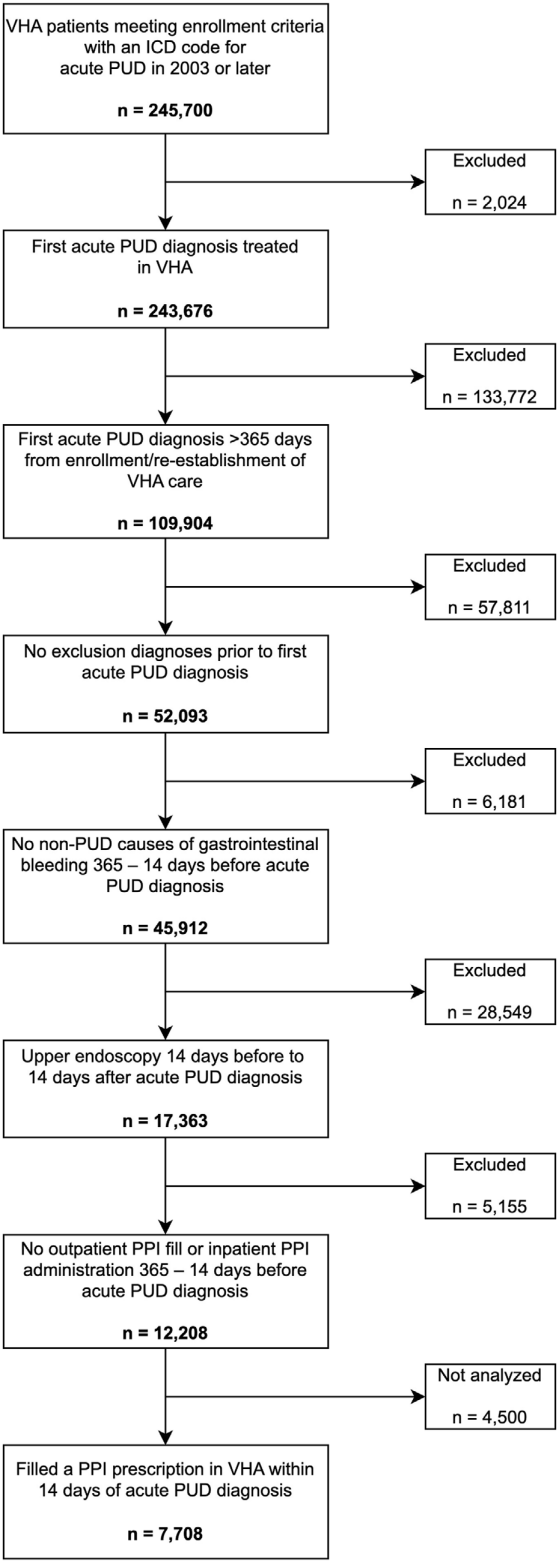
Cohort eligibility flowchart.

We identified 12 208 patients with an acute PUD diagnosis who met cohort eligibility criteria, of whom 7708 (63%) filled a prescription for PPI in the VHA within 14 days (cohort attrition diagram presented in Figure 2). The median age was 79 (IQR 71–85), 7% were female, and 18% had their PUD diagnosis during an inpatient hospitalization. Additional patient characteristics are presented in Table 1 and Table S3.

**TABLE 1:** Patient characteristics.

	Acute PUD cohort	Acute PUD and filled a PPI prescription in VHA within 14 days
	*n* = 12 293	*n* = 7708
Age (Median, IQR)	79 (72–85)	79 (71–85)
Female (%)	845 (7%)	572 (7%)
Race (%)		
Indigenous American or Alaska Native	135 (1%)	83 (1%)
Asian	101 (1%)	63 (1%)
Black	2322 (19%)	1416 (18%)
Native Hawaiian or Pacific Islander	120 (1%)	75 (1%)
White	8692 (71%)	5556 (72%)
Declined/Unknown race	838 (7%)	515 (7%)
Missing race	85 (1%)	0 (0%)
Ethnicity (%)		
Not Hispanic	11 111 (90%)	7043 (91%)
Hispanic	576 (5%)	348 (5%)
Declined/Unknown ethnicity	521 (4%)	317 (4%)
Missing ethnicity	85 (1%)	0 (0%)
Inpatient PUD diagnosis (%)	3234 (26%)	1352 (18%)
PUD diagnosis with gastrointestinal bleeding	921 (7%)	400 (5%)
Charlson‐Deyo score (median, IQR)	2 (1–5)	2 (1–4)
VA Frailty Index (%)		
Nonfrail	2749 (22%)	1863 (24%)
Prefrail	5110 (42%)	3341 (43%)
Mildly frail	2842 (23%)	1695 (22%)
Moderately frail	1100 (9%)	592 (8%)
Severely frail	492 (4%)	217 (3%)
Concurrent prescriptions (Median, IQR)	6 (3–10)	6 (3–10)
Usual Provider of Care index (Median, IQR)	0.80 (0.58–0.92)	0.80 (0.58–0.92)

Among the 7708 patients who filled a PPI in the VHA, 2717 (35%) had at least one filled PPI prescription exceeding the approved duration. The median number of prescriptions exceeding the approved duration was 3 (IQR 2–4) for 346 median days of excess PPI exposure (IQR 165–643) (additional descriptive statistics in Data S1). Forty‐four percent of filled PPI prescriptions exceeding approved duration were prescribed by primary care physicians, 18% were prescribed by residents/fellows, 16% were prescribed by advanced practice providers, and 16% were prescribed by gastroenterologists (Table S4). Results were similar in the secondary analysis accounting for gastroprotection guidelines, as 2080 patients (27%) had at least one filled PPI prescription exceeding the approved duration (median 3 prescriptions per patient [IQR 2–4] for 319 median days of excess PPI exposure [IQR 148–626]).

### 3.1 Factors Associated With a Filled PPI Prescriptions Exceeding the Approved Treatment Duration

In the final multivariable model, age, inpatient PUD diagnosis, VA Frailty Index, number of concurrent prescriptions at PUD diagnosis, anticoagulants, aspirin, and NSAIDs were associated with a filled PPI prescription exceeding the approved treatment duration, whereas female sex, Black race, and calendar year were inversely associated (Table 2, Table S5). The strongest identified associations were inpatient PUD diagnosis (aHR 1.32, 95% CI 1.25–1.39), NSAIDs (aHR 1.26, 95% CI 1.18–1.34), anticoagulants (aHR 1.25, 95% CI 1.13–1.38), Black race (aHR 0.83, 95% CI 0.78–0.88), and moderately frail VA Frailty Index (1.15, 95% CI 1.06–1.26). The final multivariable model for the secondary outcome accounting for gastroprotection guidelines was similar except that female sex was not associated (Table 2).

**TABLE 2:** Factors associated with filled PPI prescriptions exceeding the approved treatment duration in the final multivariable Andersen‐Gill recurrent event model.

	Primary outcome: Filled PPI prescription exceeding the approved treatment duration		Secondary outcome: Filled PPI prescription exceeding the approved treatment duration accounting for gastroprotection guidelines	
	aHR	95% CI	aHR	95% CI
Age	1.00	1.00–1.01	1.01	1.00–1.01
Female sex	0.91	0.83–0.99	—	
Race (ref: White)				
Indigenous American or Alaska Native	1.02	0.84–1.24	1.12	0.89–1.40
Asian	0.97	0.74–1.26	0.94	0.69–1.30
Black	0.83	0.78–0.88	0.81	0.76–0.86
Native Hawaiian or Pacific Islander	1.13	0.95–1.34	1.03	0.82–1.29
Declined/Unknown race	1.01	0.93–1.10	1.04	0.95–1.13
Acute PUD diagnosis calendar year	0.98	0.98–0.99	0.99	0.98–0.99
Inpatient acute PUD diagnosis	1.32	1.25–1.39	1.34	1.26–1.42
VA frailty index (ref: Nonfrail)				
Prefrail	1.06	1.00–1.12	1.07	1.01–1.14
Mildly frail	1.11	1.04–1.18	1.12	1.04–1.20
Moderately frail	1.15	1.06–1.26	1.12	1.01–1.24
Severely frail	1.08	0.94–1.23	1.12	0.96–1.31
Concurrent prescriptions	1.01	1.01–1.01	1.01	1.01–1.01
Time‐varying factors				
Anticoagulants	1.25	1.13–1.38	1.18	1.04–1.33
Aspirin	1.15	1.07–1.24	1.14	1.04–1.24
NSAIDs	1.26	1.18–1.34	1.16	1.02–1.32

*Note:* — indicates that the variable was not associated with the parsimonious final model.

### 3.2 Adjusted Population Attributable Fraction

**FIGURE 3:**
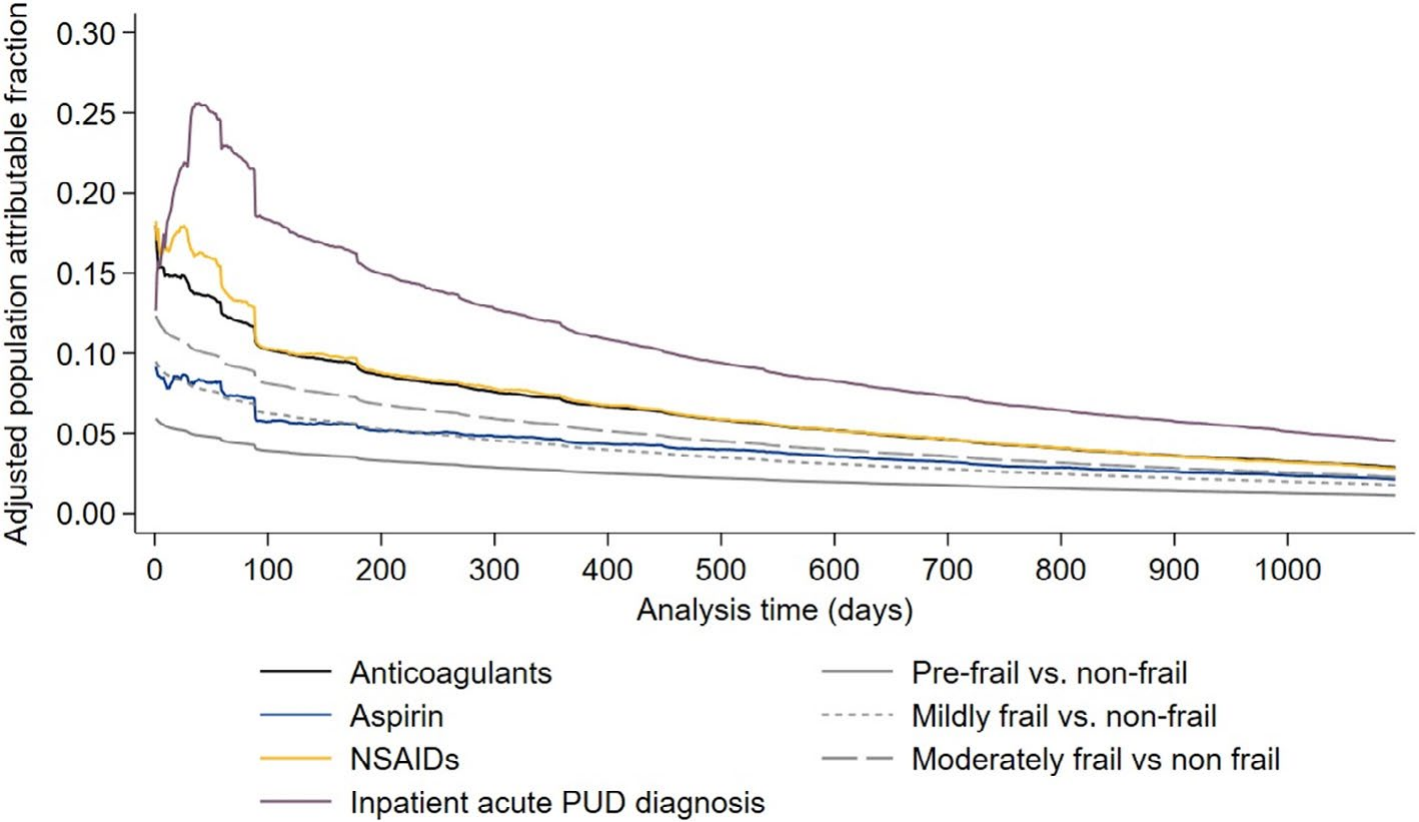
Adjusted population‐attributable fraction for selected factors positively associated with PPI prescriptions exceeding the approved treatment duration (primary outcome). The population‐attributable fraction represents the proportion of PPI prescriptions that would be eliminated if the factor were not present in the population.

On the health‐system level, inpatient PUD diagnosis had the highest adjusted population attributable fraction for filled PPI prescriptions exceeding the approved treatment duration for the majority of follow‐up, peaking at 0.26 39 days after PUD diagnosis. The peak adjusted population attributable fractions for NSAIDS, anticoagulants, and aspirin were 0.18, 0.18, and 0.09, respectively. The peak‐adjusted population‐attributable fraction of VA Frailty Index levels relative to nonfrail was 0.06 for prefrail, 0.09 for mildly frail, and 0.12 for moderately frail (Figure 3). The adjusted population‐attributable fractions for the secondary outcome accounting for gastroprotection guidelines were nearly identical to those for the primary outcome (Figure S3).

### 3.3 Sensitivity Analysis

The results of the four sensitivity analyses related to follow‐up time, delayed gastric ulcer healing, PPI‐antiplatelet drug–drug interactions, and the VA Frailty Index were consistent with the main analysis with respect to the most strongly associated factors (Table S6).

## 4 Discussion

In this retrospective cohort study of patients with acute PUD, we found that more than one‐third of patients received at least one PPI prescription exceeding the FDA‐approved treatment duration. The median days of excess PPI exposure among these patients was nearly 1 year. On the health system level, more than one‐third of early PPI prescriptions exceeding the approved duration were attributed to inpatient PUD diagnoses and patient frailty. Additionally, nearly half of early PPI prescriptions exceeding the approved duration were attributed to NSAID, anticoagulant, or aspirin use that did not meet gastroprotection guidelines. These results may serve as evidence for the development of clinical decision support interventions that identify patients eligible for PPI deprescribing without inappropriately deprescribing PPIs for patients with indications for gastroprotection.

The results of this study indicate that patient complexity is a driver for PPI persistence when it is no longer clinically indicated. For example, the peak adjusted population attributable fraction of an inpatient PUD diagnosis was 0.26—indicating that if patients with inpatient PUD diagnosis were treated equivalently to patients with outpatient PUD diagnoses, 26% of PPI prescriptions exceeding the approved duration would be eliminated from the health system. Furthermore, an additional 12% were attributed to patient frailty. An explanation for these findings could be that patients with multiple complex chronic conditions often have several issues that need to be assessed per encounter, leading to provider overload and inertia for managing conditions that are quiescent, such as treated PUD [44, 45, 46]. This study also highlights how the status quo for PPI deprescribing could result in harm. Black patients had 28% lower rates and female patients had 9% lower rates of PPI prescriptions exceeding the approved treatment duration. Although in this case, the difference results in protection from inappropriate medical care, it highlights how the current opportunistic deprescribing paradigm has the potential to exacerbate healthcare inequities. Similar health disparities have been demonstrated in other settings for receipt of guideline‐concordant and guideline‐discordant care [47, 48].

To avoid these consequences of opportunistic deprescribing, health system‐wide deprescribing efforts are needed. For example, a PPI deprescribing intervention implemented in VHA facilities in the U.S. Southwest in 2013 restricted prescription duration for patients who did not have appropriate provider‐entered long‐term PPI indications. While this intervention reduced PPI utilization, it also discontinued appropriate PPI prescriptions for patients at risk for upper gastrointestinal bleeding [20]. Our study demonstrates how a more specific deprescribing program could be designed. For instance, PPI prescriptions for treatment of PUD could be defaulted to 8 weeks. Prescription renewals and refills for patients who meet gastroprotection guidelines (identified by ICD codes and concurrent prescriptions in the electronic medical record as demonstrated in this study) could be preapproved, but prescriptions for patients not meeting these criteria could be restricted until the prescriber provides an appropriate indication [49]. Such a system would allow for long‐term PPI use for approved indications while preventing inertial PPI persistence among medically complex patients who have completed PPI treatment.

This study has several strengths that allowed us to isolate factors associated with PPI prescribing. First, by focusing on acute PUD, we were able to study one of the few PPI indications that has a clear, limited treatment duration and is ascertainable in administrative health data. Second, by using data from the VHA, we were able to reduce the impact of PPI exposure misclassification from patients using over‐the‐counter PPIs, as VHA medication copays range from $0 – $8 for 30‐day supplies of PPIs, which is significantly lower than over‐the‐counter prices of generic PPI formulations in the United States. Furthermore, more than 80% VHA refill and reorder prescriptions are fulfilled through mail order, thereby reducing the likelihood of confounding by patient proximity to VHA healthcare facilities [50]. Third, using a cohort study design with time‐to‐event analysis, we were able to leverage longitudinal data from each patient to assess time‐varying factors, such as anticoagulant and antiplatelet use, and censor patients who developed new indications for PPIs, such as gastroesophageal reflux disease. Fourth, the main study results were confirmed in four sensitivity analyses that explored the impact of key study design assumptions.

This study also has limitations. First, as this cohort predominately included Veterans over age 65, many patients may have been dual insured by the VHA and Medicare. Although we did not assess Medicare data directly in this study, we limited the effects of potential misclassification by studying a cohort of Veterans who highly utilized the VHA for their longitudinal health care and PUD management. Second, the factors associated with PPI persistence in this cohort of predominately male patients who are older than age 65 may not generalize to other populations. However, as older adults are at the highest risk for reported PPI complications, the conclusions of this study are relevant on a population level. Third, as this is an observational study, it is possible that there were unmeasured confounders that influenced the reported associations. Fourth, the final sample size of 7708 patients was relatively small for a nationwide study using two decades of VHA data. This was a consequence of the strict eligibility criteria employed to reduce misclassification of the study exposures, outcomes, and covariates. Broadening the eligibility criteria may have introduced confounding to the study results from patients with unrecognized indications for long‐term PPI use.

In conclusion, this study demonstrates that patient factors signifying medical complexity influence PPI prescribing beyond the FDA‐approved treatment duration for acute PUD after adjustment for patient, provider, and facility factors. Because long‐term PPI use is potentially associated with adverse effects and unnecessary healthcare expenses, the conclusions of this study may inform future deprescribing interventions that effectively, efficiently, and equitably identify patients least likely to benefit from long‐term PPI use. Furthermore, the methodology employed in this study may serve as a model to develop foundational deprescribing data for other medications that are overused, thereby facilitating future targeted deprescribing paradigms.

### 4.1 Plain Language Summary

Proton pump inhibitors (PPIs) are used to treat peptic ulcer disease (PUD), which are ulcers that occur in the stomach or the small intestine. Regulatory agencies recommend that PPIs be used for no more than 8 weeks, and recent studies suggest that long‐term PPI use is associated with side effects like intestinal infection and reduced kidney function. We used patient data from the Veterans Health Administration in the United States to determine rates of PPI treatment for PUD exceeding the eight‐week treatment duration and healthcare factors associated with this overuse. We identified that 35% of patients treated for PUD had PPI use exceeding the approved treatment duration. Markers of patient complexity, such as being diagnosed with PUD as an inpatient and use of medications that can potentially cause gastrointestinal bleeding, were associated with PPI prescriptions exceeding the approved treatment duration. These results may inform the development of deprescribing programs that assist prescribers and health systems in reducing inappropriate long‐term PPI use.

## Author Contributions

Conceptualization: Jordan A. Villars, Ravy K. Vajravelu. Methodology: Jordan A. Villars, Timothy S. Anderson, Ravy K. Vajravelu. Software: N/A. Validation: N/A. Formal analysis: Ravy K. Vajravelu. Investigation: All authors. Resources: Ravy K. Vajravelu. Data curation: Ravy K. Vajravelu. Writing – original draft: Jordan A. Villars, Ravy K. Vajravelu. Writing – review and editing: All authors. Visualization: Villars, Ravy K. Vajravelu. Supervision: Ravy K. Vajravelu. Project administration: Ravy K. Vajravelu. Funding acquisition: Ravy K. Vajravelu. The corresponding author attests that all listed authors meet authorship criteria and that no others meeting the criteria have been omitted.

## Disclosure

T.A.S. and R.K.V. are employees of the Department of Veterans Affairs. This research does not necessarily represent the views of the Department of Veterans Affairs or the United States Government. T.A.S. reports receiving research grants from the American Heart Association, the American College of Cardiology, and the U.S. Deprescribing Research Network; travel grants from the Society for Hospital Medicine and American College of Physicians; and personal fees from the American Medical Student Association and American Medical Association—all unrelated to this research. R.E.S. reports research support from Exact Sciences, Freenome, and Immunovia and advisory support from Guardant—all unrelated to this research. J.A.V. and J.G.Y. report no disclosures.

## Conflicts of Interest

The authors declare no conflicts of interest.

## Supporting information


**Data S1.** Supporting Information.

**Data S2.** Supporting Information.

## Data Availability

Statistical programming code used to generate the study results are available on the corresponding author's GitHub repository at https://github.com/rvajravelu/inappropriatePpi with a README file describing the function of each file. Raw data are available to investigators with data use agreements with the Department of Veterans Affairs to access the Veterans Health Administration Corporate Data Warehouse.
