# Supplementary material for: Proton Pump Inhibitor Use Exceeding the U.S. Food and Drug Administration Approved Treatment Duration for Patients With Peptic Ulcer Disease: A Retrospective Cohort Study
Source: Pharmacoepidemiol Drug Saf. 2025 Apr 29;34(5):e70152. doi: 10.1002/pds.70152 (PMC12038380; doi:10.1002/pds.70152)

**SUPPLEMENTAL MATERIAL**

Proton Pump Inhibitor Use Exceeding the U.S. Food and Drug Administration Approved

Treatment Duration for Patients with Peptic Ulcer Disease: A Retrospective Cohort Study

Jordan A. Villars MD MPA, Timothy S. Anderson MD MAS, Jonathan G. Yabes PhD, Robert E.

Schoen MD MPH, Ravy K. Vajravelu MD MSCE

**Contents (hyperlinks):**

**[Supplemental methods ................................................................................................................ 3](#br3)**

*[Cohort eligibility: Acute PUD diagnosis (additional details)....................................................................3](#br3)*

*[Aspirin, antiplatelet, anticoagulant, NSAID, and H2-receptor antagonist exposure ascertainment .............4](#br4)*

*[Statistical analysis (model building details and handling of missing data) ...............................................4](#br4)*

*[Statistical analysis (adjusted population attributable fraction)...............................................................5](#br5)*

**[Supplemental results ................................................................................................................... 6](#br6)**

**[Supplemental references............................................................................................................. 7](#br7)**

**[Supplemental table 1: ICD codes for acute PUD ...................................................................... 8](#br8)**

**[Supplemental table 2. List of anticoagulants, antiplatelets, NSAIDS, and H2-receptor](#br10)**

**[antagonists.................................................................................................................................. 10](#br10)**

**[Supplemental table 3. Patients not censored by days from acute PUD diagnosis ............ 11](#br11)**

**[Supplemental table 4. Number and rates of PPI prescriptions with exceeding the](#br12)**

**[approved treatment duration by provider type ....................................................................... 12](#br12)**

**[Supplemental table 5. Factors associated with PPI prescriptions exceeding the approved](#br14)**

**[treatment duration in univariable Andersen-Gill recurrent event models........................... 14](#br14)**

**[Supplemental table 6. Sensitivity analysis results for the primary outcome...................... 16](#br16)**

**[Supplemental table 7: ICD codes for chronic or unspecified PUD. Exclusions any time](#br18)**

**[before first acute PUD diagnosis.............................................................................................. 18](#br18)**

**[Supplemental table 8. ICD codes for indications for PPI use, upper gastrointestinal](#br20)**

**[malignancy, and altered upper gastrointestinal anatomy. Exclusions applied any time](#br20)**

**[before first PUD diagnosis. ....................................................................................................... 20](#br20)**

**[Supplemental table 9. ICD codes for non-PUD causes of GI bleeding. Exclusions applied](#br23)**

**[365 – 14 days before the first acute PUD diagnosis............................................................... 23](#br23)**

**[Supplemental table 10. Current Procedural Terminology-4 codes for upper endoscopy . 24](#br24)**

**[Supplemental figure 1. Conceptual model for relationship of assessed healthcare factors](#br26)**

**[with filled PPI prescriptions exceeding the approved treatment duration .......................... 26](#br26)**

[(Return to contents)](#br1)


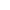


JA Villars et al.

2

**[Supplemental figure 2. Assessment intervals for cohort eligibility, outcome, and factor](#br27)**

**[criteria .......................................................................................................................................... 27](#br27)**

**[Supplemental figure 3. Adjusted population-attributable fraction for selected factors](#br29)**

**[positively associated with PPI prescriptions exceeding the approved treatment duration](#br29)**

**[(secondary outcome). ................................................................................................................ 29](#br29)**

[(Return to contents)](#br1)


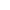


JA Villars et al.

3

**Supplemental methods**

*Cohort eligibility: Acute PUD diagnosis (additional details)*

Among patients enrolled in the VHA, we excluded those with less than 365 days from

their first inpatient/outpatient encounter to their last encounter to ensure sufficient data to

identify pre-existing conditions that contribute to the exclusion criteria and covariates. We also

classified periods in which a patient had more than 365 days between VHA encounters as gaps

in utilization. Encounters occurring after age 90 were censored to reduce reidentification risks.

Patients who had their first acute PUD diagnosis before 2003 (one year after nationwide

implementation of electronic health records in the VHA) or had their PUD diagnosed in a non-

VHA facility were excluded to reduce misclassification of dates and covariates. To identify

patients with an incident diagnosis of acute PUD as opposed to recurrent or chronic PUD, we

stipulated that the PUD diagnosis should occur at least 365 days after the first VHA encounter

and at least 365 days after any gap in VHA utilization.^1^ Additionally, we excluded patients with

ICD codes for chronic or unspecified PUD prior to the first acute PUD diagnosis (**Supplemental**

**table 7**). Because the outcome of interest was filled PPI prescriptions exceeding the approved

treatment duration for PUD, we excluded patients with other indications for chronic PPI use

before the acute PUD diagnosis, such as gastroesophageal reflux disease, Barrett’s esophagus,

and eosinophilic esophagitis. Additionally, because upper gastrointestinal malignancies and

surgically altered upper gastrointestinal anatomy may influence PPI prescribing, we excluded

patients with these diagnoses prior to the acute PUD diagnosis (**Supplemental table 8**).

Furthermore, because non-PUD causes of gastrointestinal bleeding could influence PPI

prescribing, patients with ICD codes for conditions such as hematemesis, angiodysplasia of the

intestine, and melena were excluded if they experienced the alternative cause of bleeding 365 –

14 days before the acute PUD diagnosis (**Supplemental table 9**). The 14-day gap between the

end of the exclusion period and the acute PUD diagnosis was implemented to allow for

[(Return to contents)](#br1)


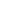

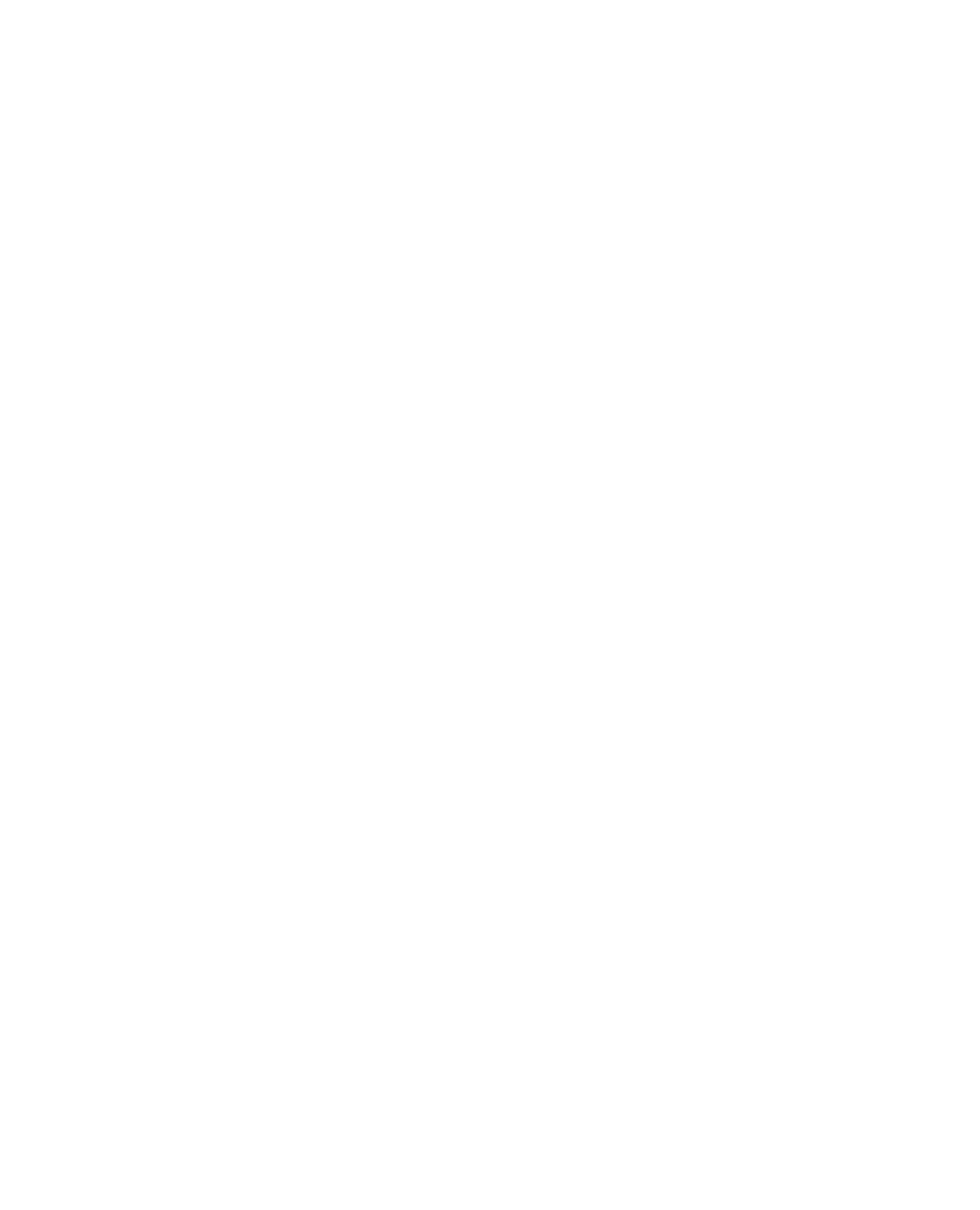


JA Villars et al.

4

prodromal symptoms of acute PUD. Next, to limit the cohort to patients most likely to have acute

PUD, we excluded patients who did not have an upper endoscopy 14 days before – 14 days

after the first acute PUD diagnosis. This 28-day range was implemented to allow for delays in

PUD diagnosis code entry after an upper endoscopy that identified PUD and delays from

suspected PUD diagnosis to confirmatory upper endoscopy. Upper endoscopy was identified

using Current Procedural Terminology-4 codes (**Supplemental table 10**). Finally, to exclude

patients with indications for chronic PPI that were not captured by ICD codes, patients with filled

prescriptions for PPIs in the 365 – 14 days before the first acute PUD diagnosis were excluded.

*Aspirin, antiplatelet, anticoagulant, NSAID, and H2-receptor antagonist exposure ascertainment*

Exposure to each medication class was ascertained through prescription fills to develop

time-updating indicator variables. Using the days supplied, we calculated start and end dates for

prescriptions. When new prescriptions were filled prior to completion of the prior prescription,

the start and end dates were delayed based on the backstock of medication from the prior

prescriptions.^2^

*Statistical analysis (model building details and handling of missing data)*

To develop a parsimonious, well-fitting multivariable model with minimal collinearity

among factors, we first assessed the univariable association of each factor with a filled PPI

prescription exceeding the approved treatment duration. Factors with Wald test p-values < 0.15

were considered for the multivariable model. Because the Charlson-Deyo score and the VA

Frailty Index are both measures of comorbidities, we assessed them separately and together to

determine whether models with Charlson-Deyo alone, VA Frailty Index alone, or both Charlson-

[(Return to contents)](#br1)


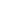

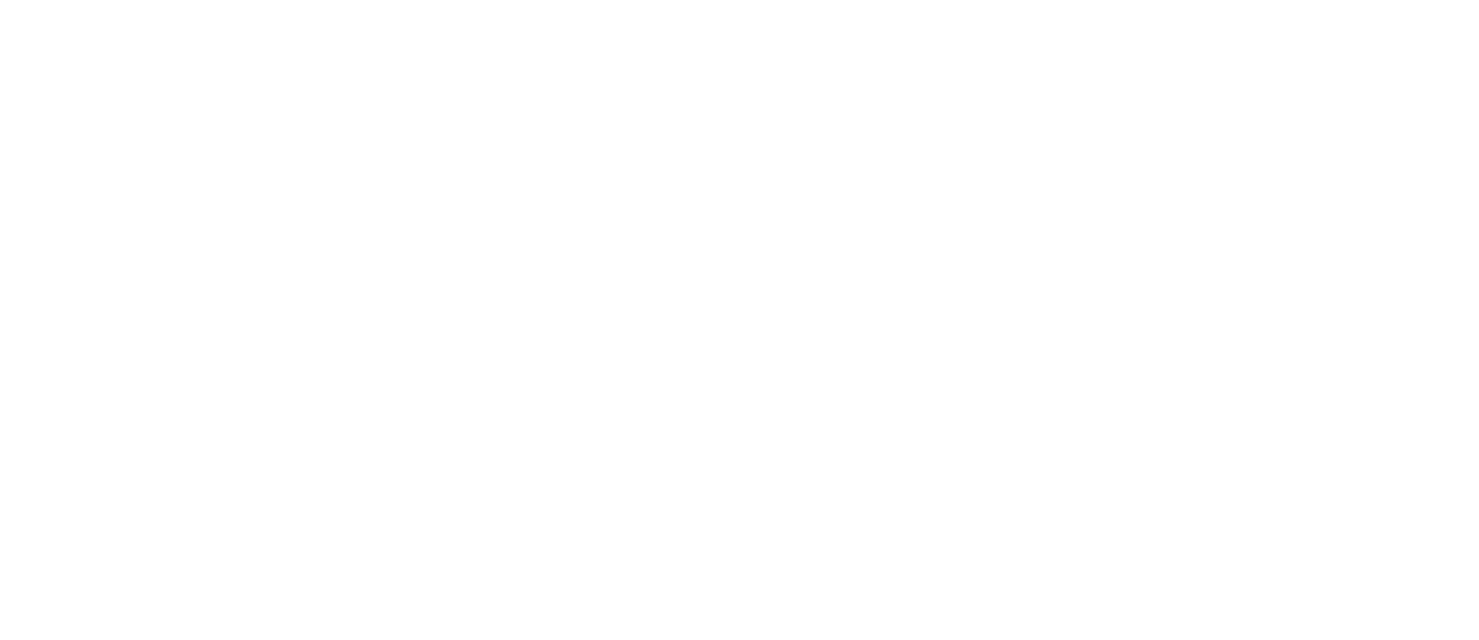

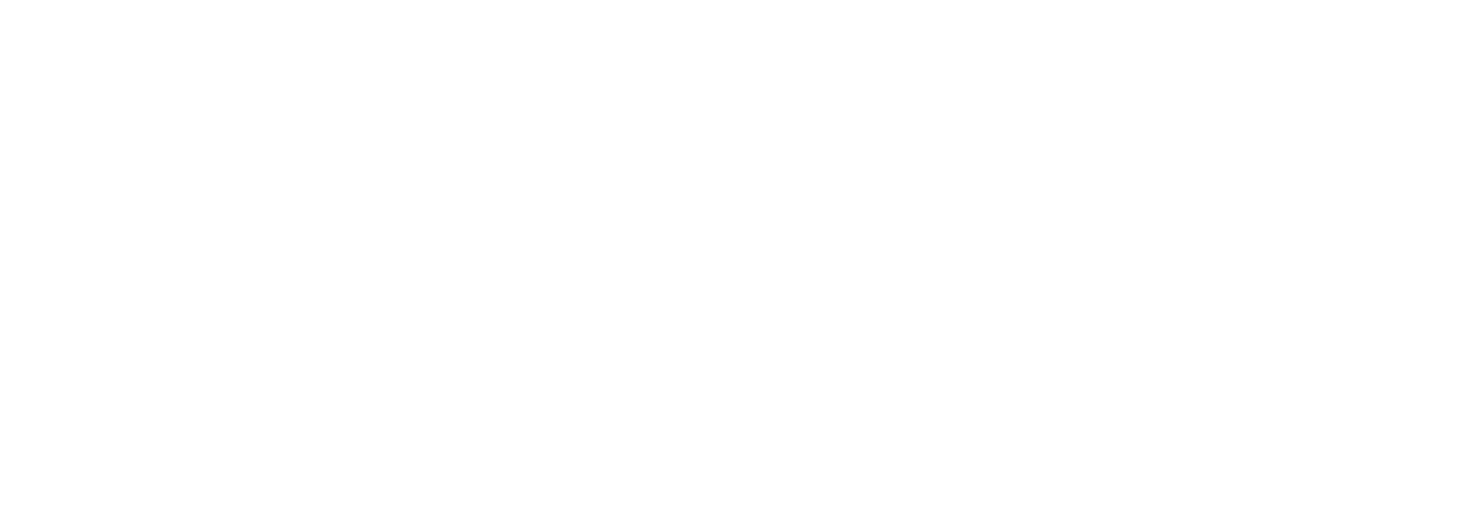

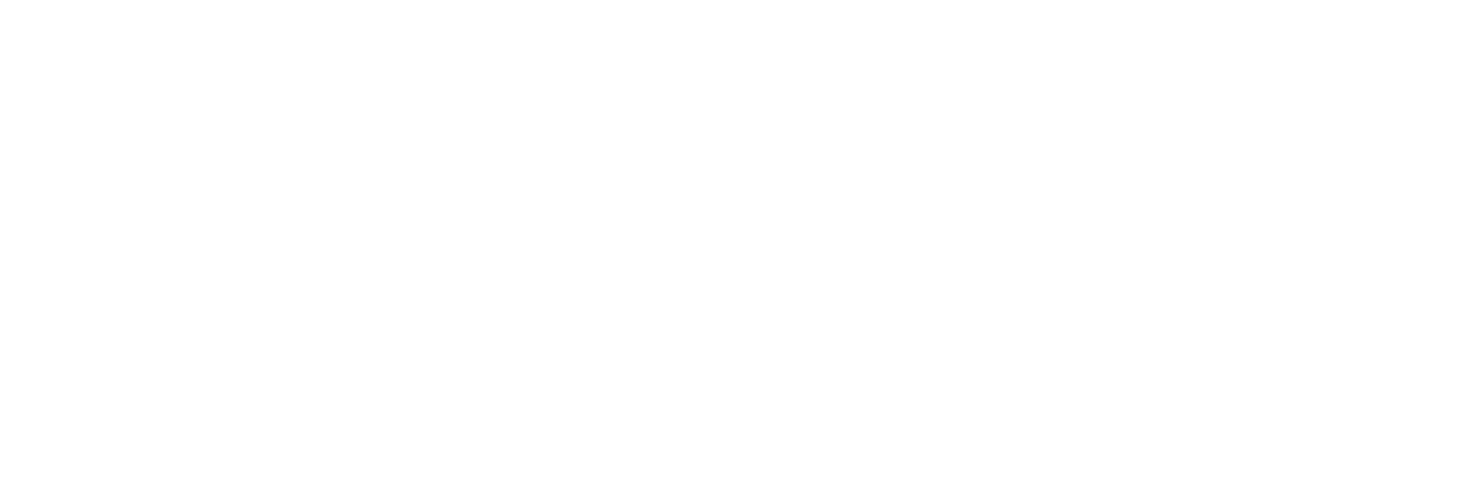


JA Villars et al.

5

Deyo and VA Frailty Index were most explanatory. The best fitting model of these three was

determined by the lowest Bayesian Information Criterion. Factors with p-values < 0.15 were

then input into a multivariable model. Additional candidate multivariable models were developed

by sequentially eliminating factors with Wald p-values ≥ 0.05. The final model was selected by

choosing the multivariable model with the lowest Bayesian Information Criterion, even if this

model did not have only factors with Wald p-values < 0.05. Adherence to the proportional

hazards assumption was assessed using Schoenfeld residuals and complementary log-log

survival plots.

Patients with missing date of birth, sex, or VHA enrollment information were excluded

from the cohort as this is a marker of poor data quality. Because the exposures, outcomes, and

health factors were derived from administrative health data, absence of the respective codes

indicates that the patient did not experience the covariate. As such, there was no missingness in

these covariates among the patients in the cohort.

*Statistical analysis (adjusted population attributable fraction)*

The time-to-event adjusted population attributable fraction was calculated from the

cumulative incidence probability at each time point from the final multivariable Andersen-Gill

model. For each factor of interest, the attributable difference was calculated as the difference

between the cumulative incidence probability among patients who experienced the factor minus

the counterfactual cumulative incidence probability among patients who did not experience the

factor. For time varying factors, the counterfactual cumulative incidence probability was

calculated by censoring patients at the time they experienced the factor. To calculate the

[(Return to contents)](#br1)


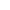

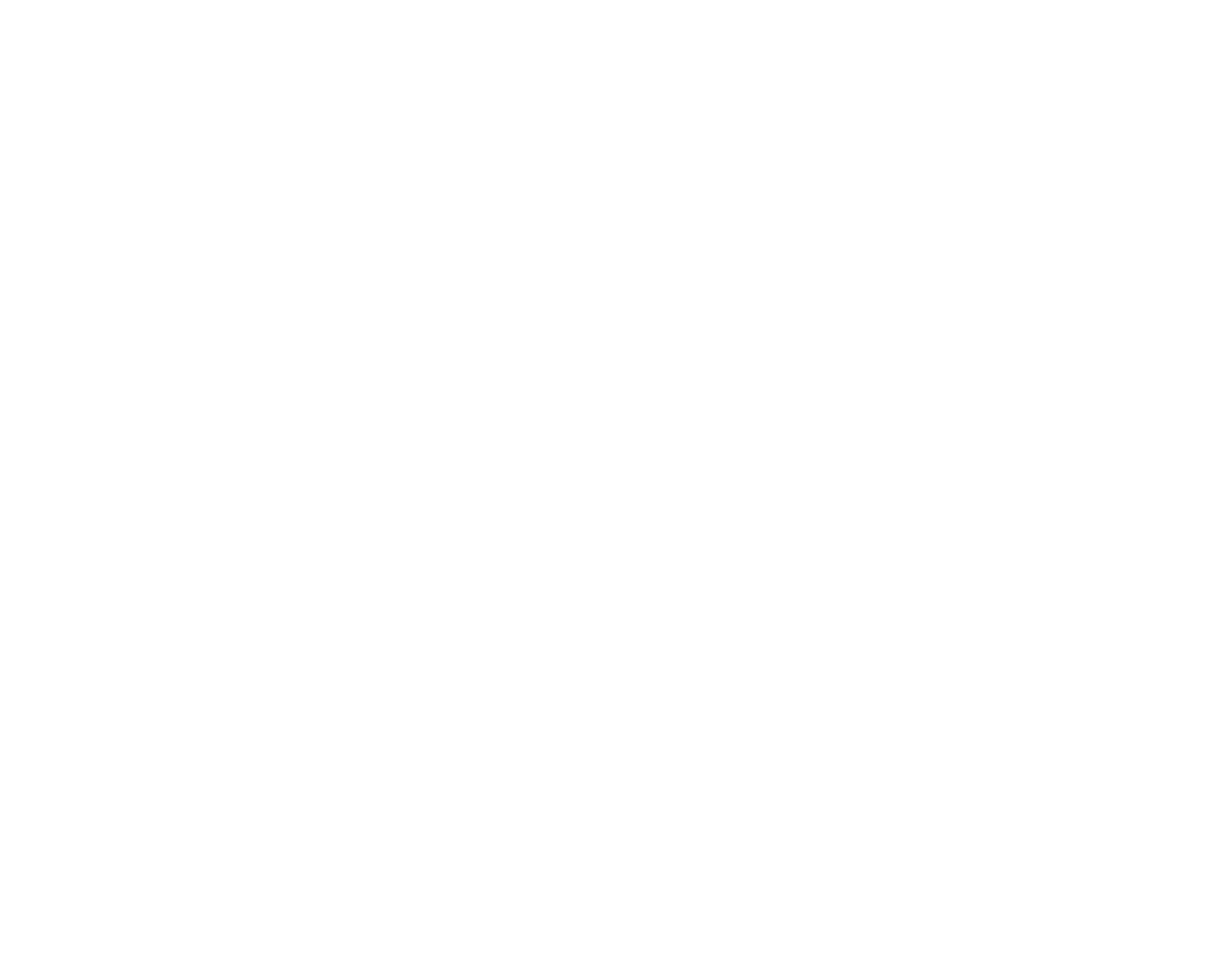

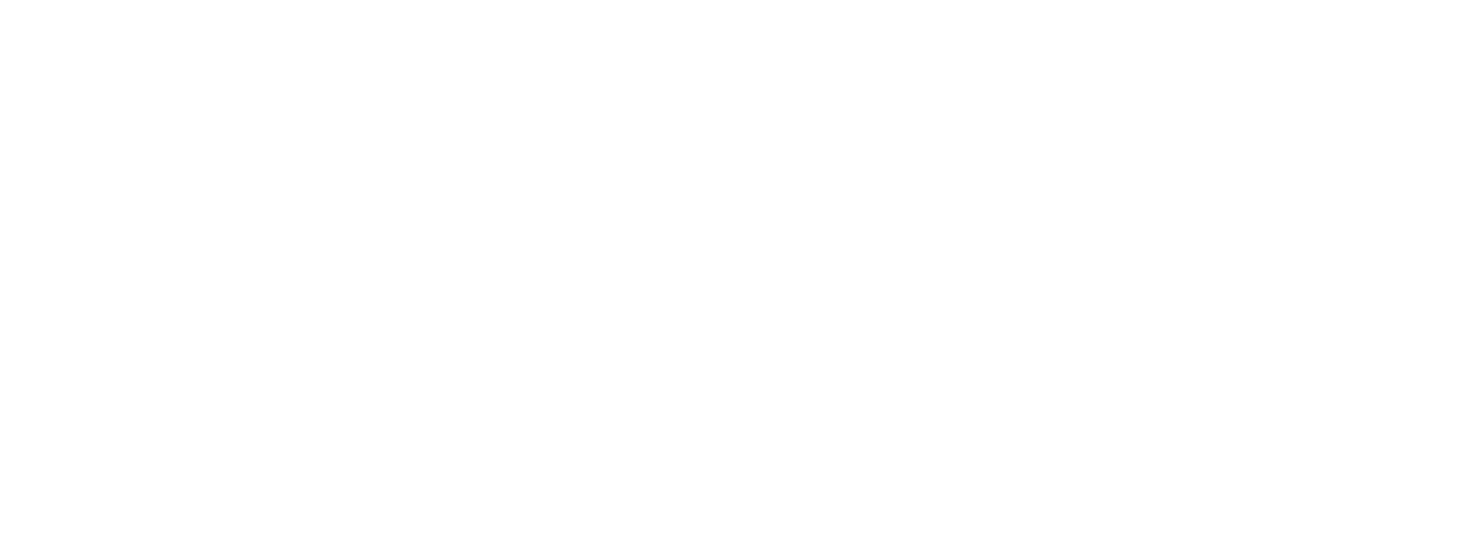


JA Villars et al.

6

population attributable fraction, the attributable difference was divided by the cumulative

incidence probability among patients who experienced the factor.^3^

**Supplemental results**

In total there were 11,289 filled PPI prescriptions exceeding the approved treatment

duration among the 7,708 patients. Per patient, the median number of PPI prescriptions

exceeding the approved treatment duration was 1 (IQR 1 – 2). Among the 7,708 patients who

filled a PPI in the VHA, 2,717 (35%) had at least one filled PPI prescription exceeding the

approved treatment duration. Among these patients, the median number of prescriptions with

exceeding the approved treatment duration was 3 (IQR 2 – 4) for a median number of days of

excess PPI exposure of 346 (IQR 165 – 643). The maximum number of filled PPI prescriptions

exceeding the approved treatment duration was 13. The maximum days of excess PPI

exposure was 1,034.

[(Return to contents)](#br1)


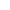

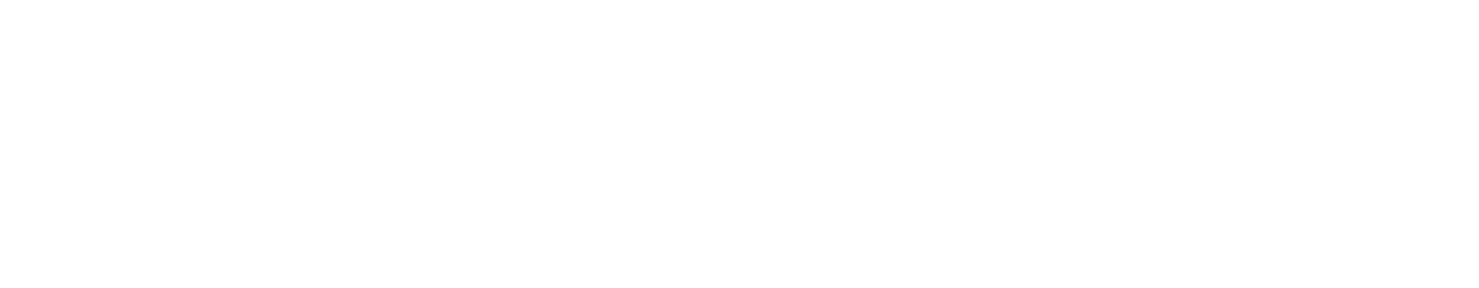

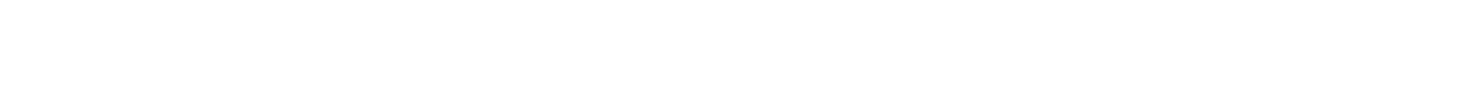


JA Villars et al.

7

**Supplemental references**

1.

Lewis JD, Bilker WB, Weinstein RB, Strom BL. The relationship between time since

registration and measured incidence rates in the General Practice Research Database.

*Pharmacoepidemiol Drug Saf*. Jul 2005;14(7):443-51. doi:10.1002/pds.1115

2.

Thai TN, Winterstein AG. Core concepts in pharmacoepidemiology: Measurement of

medication exposure in routinely collected healthcare data for causal inference studies in

pharmacoepidemiology. *Pharmacoepidemiol Drug Saf*. Mar 2024;33(3):e5683.

doi:10.1002/pds.5683

3.

von Cube M, Schumacher M, Timsit JF, Decruyenaere J, Steen J. The population-

attributable fraction for time-to-event data. *Int J Epidemiol*. Jun 6 2023;52(3):837-845.

doi:10.1093/ije/dyac217

4.

Guerin A, Mody R, Carter V, et al. Changes in Practice Patterns of Clopidogrel in

Combination with Proton Pump Inhibitors after an FDA Safety Communication. *PLoS One*.

2016;11(1):e0145504. doi:10.1371/journal.pone.0145504

[(Return to contents)](#br1)


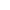

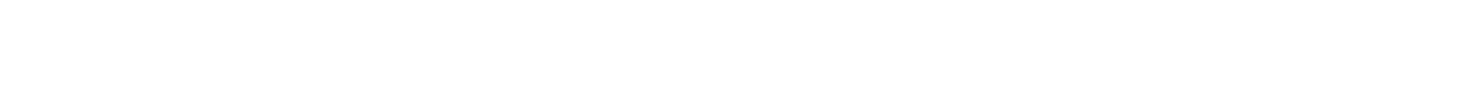


JA Villars et al.

8

**Supplemental table 1: ICD codes for acute PUD**

**Code**

**Description**

**Type**

ICD9

ICD9

ICD9

ICD9

531.00

531.01

531.20

531.21

Acute gastric ulcer with hemorrhage, without mention of obstruction

Acute gastric ulcer with hemorrhage, with obstruction

Acute gastric ulcer with perforation, without mention of obstruction

Acute gastric ulcer with perforation, with obstruction

Acute gastric ulcer with hemorrhage and perforation, without mention of

obstruction

532.00

532.01

ICD9

ICD9

Acute gastric ulcer with hemorrhage and perforation, with obstruction

Acute gastric ulcer without mention of hemorrhage or perforation, without

mention of obstruction

Acute gastric ulcer without mention of hemorrhage or perforation, with

obstruction

532.20

532.21

ICD9

ICD9

Gastric ulcer, unspecified as acute or chronic, without mention of

hemorrhage or perforation, without mention of obstruction

533.00

533.01

533.20

ICD9

ICD9

ICD9

Acute duodenal ulcer with hemorrhage, without mention of obstruction

Acute duodenal ulcer with hemorrhage, with obstruction

Acute duodenal ulcer with hemorrhage and perforation, without mention of

obstruction

533.21

531.10

ICD9

ICD9

Acute duodenal ulcer with hemorrhage and perforation, without obstruction

Acute duodenal ulcer with hemorrhage and perforation, without mention of

obstruction

531.11

531.30

ICD9

ICD9

Acute duodenal ulcer with hemorrhage and perforation, with obstruction

Acute duodenal ulcer without mention of hemorrhage or perforation, without

mention of obstruction

Acute duodenal ulcer without mention of hemorrhage or perforation, without

mention of obstruction

531.31

531.90

532.10

ICD9

ICD9

ICD9

Duodenal ulcer, unspecified as acute or chronic, without hemorrhage or

perforation, without mention of obstruction

Acute peptic ulcer of unspecified site with hemorrhage, without mention of

obstruction

532.11

532.30

ICD9

ICD9

Acute peptic ulcer of unspecified site with hemorrhage, with obstruction

Acute peptic ulcer of unspecified site with perforation, without mention of

obstruction

532.31

532.90

ICD9

ICD9

Acute peptic ulcer of unspecified site with perforation, with obstruction

Acute peptic ulcer of unspecified site with hemorrhage and perforation,

without mention of obstruction

Acute peptic ulcer of unspecified site with hemorrhage and perforation, with

obstruction

Acute peptic ulcer of unspecified site without mention of hemorrhage and

perforation, without mention of obstruction

Acute peptic ulcer of unspecified site without mention of hemorrhage and

perforation, with obstruction

Peptic ulcer of unspecified site, unspecified as acute or chronic, without

mention of hemorrhage or perforation, without mention of obstruction

533.10

533.11

533.30

533.31

533.90

ICD9

ICD9

ICD9

ICD9

ICD9

[(Return to contents)](#br1)


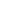

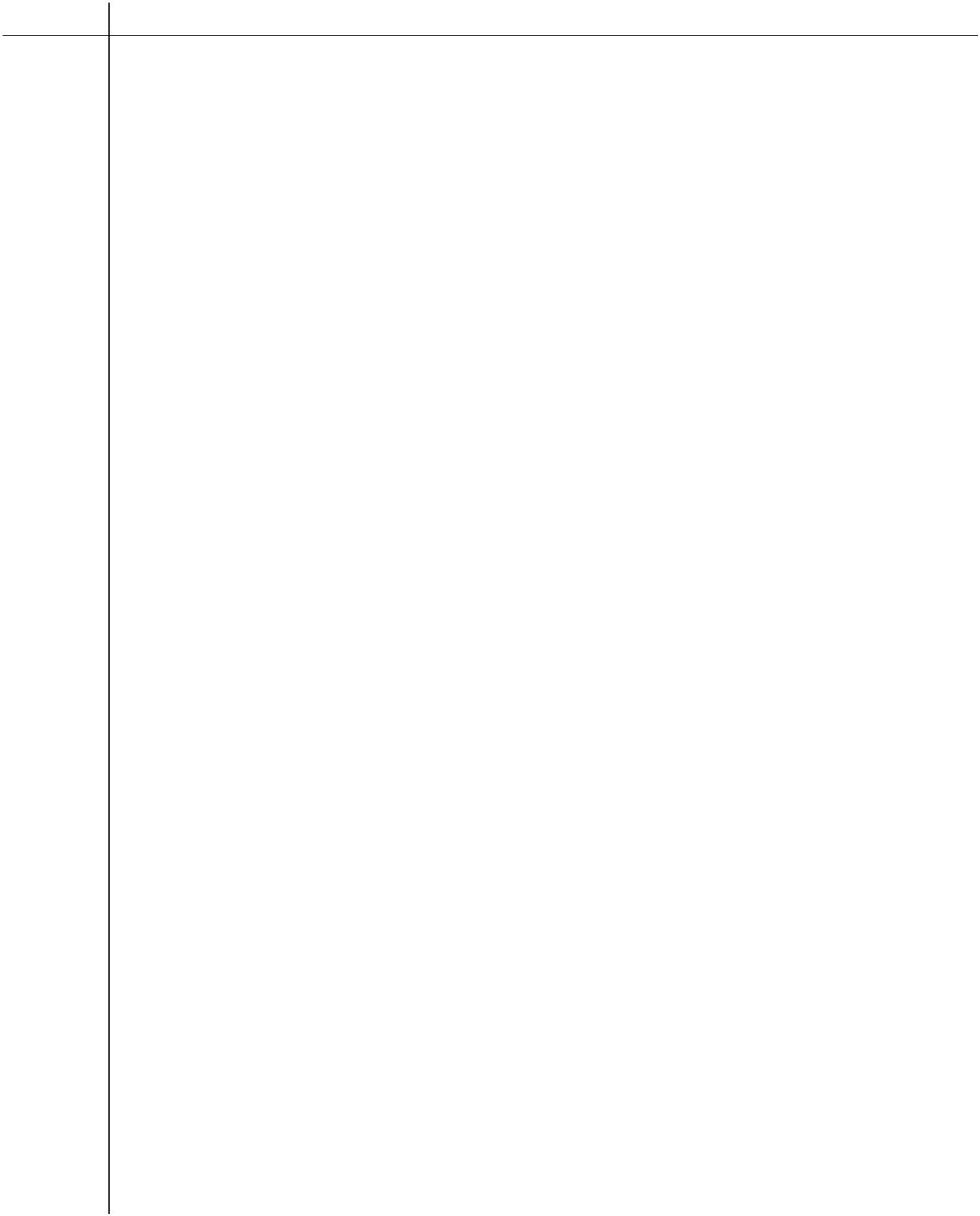


JA Villars et al.

9

**Code**

K25.0

K25.2

K26.0

K26.2

**Description**

**Type**

Acute gastric ulcer with hemorrhage

Acute gastric ulcer with perforation

Acute gastric ulcer with both hemorrhage and perforation

ICD10

ICD10

ICD10

ICD10

Acute gastric ulcer without hemorrhage or perforation

Gastric ulcer, unspecified as acute or chronic, without hemorrhage or

perforation

K27.0

K27.2

K25.1

K25.3

K25.9

ICD10

ICD10

ICD10

ICD10

ICD10

Acute duodenal ulcer with hemorrhage

Acute duodenal ulcer with perforation

Acute duodenal ulcer with both hemorrhage and perforation

Acute duodenal ulcer without hemorrhage or perforation

Duodenal ulcer, unspecified as acute or chronic, without hemorrhage or

perforation

K26.1

K26.3

K26.9

K27.1

K27.3

ICD10

ICD10

ICD10

ICD10

ICD10

Acute peptic ulcer, site unspecified, with hemorrhage

Acute peptic ulcer, site unspecified, with perforation

Acute peptic ulcer, site unspecified, with both hemorrhage and perforation

Acute peptic ulcer, site unspecified, without hemorrhage or perforation

[(Return to contents)](#br1)


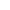

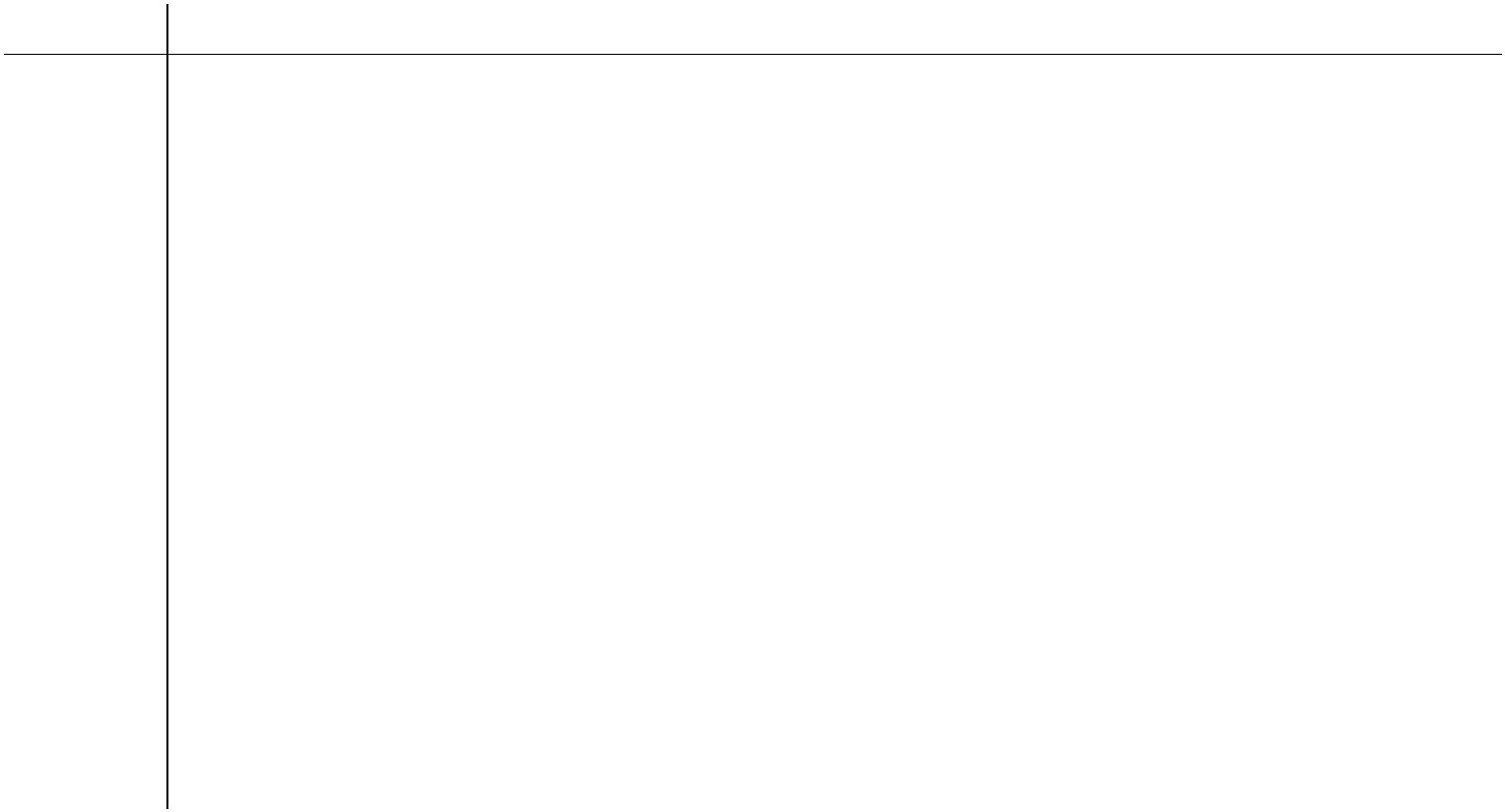


JA Villars et al. 10

**Supplemental table 2. List of anticoagulants, antiplatelets, NSAIDS, and H2-receptor**

**antagonists**

•

•

•

Antiplatelets: Clopidogrel, prasugrel, ticagrelor, ticlopidine, cilostazol, vorapaxar,

dipyridamole

Anticoagulants: Apixaban, edoxaban, rivaroxaban, dabigatran, warfarin, enoxaparin,

heparin, fondaparinux

NSAIDs: Diclofenac, diflunisal, etodolac, fenoprofen, flurbiprofen, ibuprofen,

indomethacin, ketoprofen, ketorolac, mefenam, meloxicam, nabumetone, naproxen,

oxaprozin, piroxicam, sulindac, tolmetin).

•

H2-receptor antagonists: Cimetidine, famotidine, nizatidine, ranitidine

[(Return to contents)](#br1)


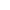


JA Villars et al. 11

**Supplemental table 3. Patients not censored by days from acute PUD diagnosis**

**Days from**

**acute PUD**

**diagnosis**

**Primary**

**outcome:**

**Filled PPI**

**prescription**

**exceeding**

**the**

**Secondary**

**outcome:**

**Filled PPI**

**prescription**

**exceeding the**

**approved**

**approved**

**treatment**

**duration**

**treatment**

**duration**

**accounting for**

**gastroprotection**

**guidelines**

0

7,708

4,367

3,557

2,919

2,405

2,032

7,708

3,366

2,596

2,061

1,639

1,342

200

400

600

800

1,000

[(Return to contents)](#br1)


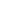

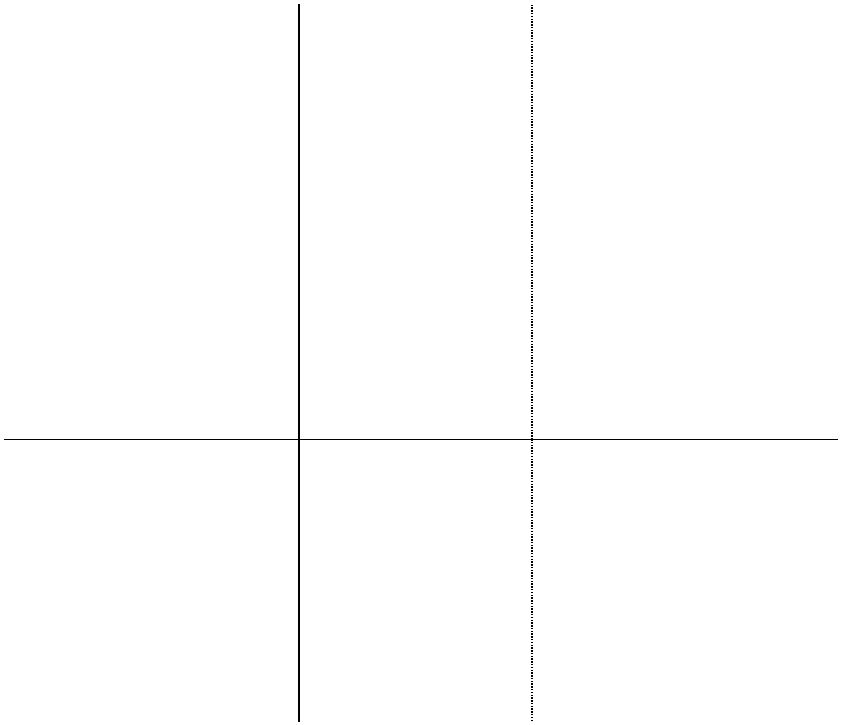


JA Villars et al. 12

**Supplemental table 4. Number and rates of PPI prescriptions with exceeding the approved treatment duration by provider**

**type**

**PPI prescriptions**

**exceeding the**

**approved**

**treatment**

**duration**

**Outpatient**

**encounters**

**Provider type**

**Ratio**

0.6

1.2

1.1

0.6

0.3

0.5

0.4

0.5

0.5

0.4

0.5

0.3

0.4

0.2

0.2

0.4

0.6

0.4

0.1

0.5

0.2

0.1

0.6

0.0

Physicians - internal medicine - no specialization

Physicians - resident, allopathic (includes interns, residents, fellows) - no specialization

Physicians - internal medicine - gastroenterology

Physicians - family medicine - no specialization

3,315

1,971

1,711

922

386

314

308

300

260

207

201

201

127

120

89

5,733

1,651

1,500

1,498

1,207

649

718

656

562

524

410

630

314

499

376

129

89

Advanced practice provider - nurse practitioner - no specialization

Advanced practice provider - physician assistant - medical

Advanced practice provider - nurse practitioner - primary care

Physicians - general practice - no specialization

Advanced practice provider - nurse practitioner - family

Advanced practice provider - nurse practitioner - adult health

Physicians - hospitalist - no specialization

Advanced practice provider - physician assistant - no specialization

Physicians - physician/osteopath - internal medicine

Physicians - surgery - no specialization

Physicians - resident, osteopathic (includes interns, residents, fellows) - no specialization

Physicians - physician/osteopath - no specialization

Physicians - family medicine - adult medicine

54

53

Physicians - internal medicine - geriatric medicine

Physicians - psychiatry & neurology - psychiatry

50

129

798

81

45

Physicians - physician/osteopath - gastroenterology

Advanced practice provider - nurse practitioner - acute care

Physicians - emergency medicine - no specialization

Physicians - family medicine - geriatric medicine

43

43

216

542

60

40

33

Missing classification - no specialization

33

2118

[(Return to contents)](#br1)


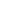

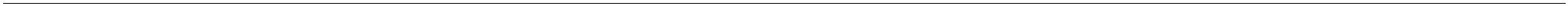


JA Villars et al. 13

**PPI prescriptions**

**exceeding the**

**approved**

**treatment**

**duration**

**Outpatient**

**encounters**

**Provider type**

**Ratio**

0.4

0.0

0.1

0.1

0.0

0.1

0.1

0.1

0.1

0.5

0.0

0.1

1.2

0.1

0.0

0.0

0.0

0.1

0.2

0.8

0.4

0.1

Advanced practice provider - nurse practitioner - gerontology

Physicians - internal medicine - cardiovascular disease

Pharmacy - pharmacist - pharmacist clinician / clinical pharmacy specialist

Physicians - emergency medicine - emergency medical services

Physicians - internal medicine - hematology & oncology

Physicians - physical medicine & rehabilitation - spinal cord injury medicine

Pharmacy - pharmacist - pharmacotherapy

27

27

23

22

22

20

19

19

19

18

17

16

15

15

15

15

15

10

8

69

797

154

345

556

145

195

243

309

35

Physicians - internal medicine - endocrinology, diabetes & metabolism

Pharmacy - pharmacist - no specialization

Physicians - internal medicine - hepatology

Physicians - radiology - diagnostic radiology

2323

187

13

Physicians - internal medicine - infectious disease

Advanced practice provider - nurse practitioner - community health

Physicians - internal medicine - rheumatology

168

331

451

532

99

Advanced practice provider - physician assistant - surgical

Physicians - internal medicine - pulmonary disease

Physicians - internal medicine - nephrology

Advanced practice provider - nurse practitioner - psychiatric/mental health

Physicians - physician/osteopath - general practice

Physicians - preventive medicine - public health & general preventive medicine

Advanced practice provider - nurse practitioner - women's health

Physicians - physician/osteopath - family practice

44

6

8

6

16

6

54

Note: Provider types with fewer than 5 PPI prescriptions not shown.

[(Return to contents)](#br1)


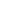

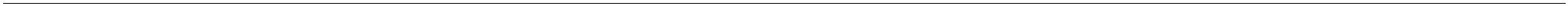


JA Villars et al. 14

**Supplemental table 5. Factors associated with PPI prescriptions exceeding the approved treatment duration in univariable**

**Andersen-Gill recurrent event models**

**Primary outcome:**

**Filled PPI prescription exceeding**

**the approved treatment duration**

**Secondary outcome:**

**Filled PPI prescription exceeding**

**the approved treatment duration**

**accounting for gastroprotection**

**guidelines**

aHR

1.01

0.84

95% CI

p

aHR

1.01

0.85

95% CI

p

Age

Female sex

1.01 – 1.01

0.77 – 0.91

0.00

0.00

1.01 – 1.01

0.76 – 0.94

0.00

0.00

Race (ref: White)

1.03

0.86

0.83

1.13

1.03

0.85 – 1.26

0.65 – 1.15

0.78 – 0.88

0.94 – 1.36

0.95 – 1.12

1.00

0.00

0.00

0.00

0.00

*Indigenous American or Alaska Native*

*Asian*

*Black*

1.13

0.87

0.81

1.01

1.05

0.90 – 1.42

0.62 – 1.24

0.76 – 0.87

0.80 – 1.28

0.96 – 1.15

0.00

0.00

0.00

1.00

0.00

*Native Hawaiian or Pacific Islander*

*Declined/Unknown race*

Ethnicity (ref: Not Hispanic)

*Hispanic*

*Declined/Unknown ethnicity*

Acute PUD diagnosis calendar year

Inpatient acute PUD diagnosis

PUD diagnosis with gastrointestinal bleeding

VA frailty index (ref: Non-frail)

*Pre-frail*

0.96

1.00

0.86 – 1.07

0.91 – 1.11

0.00

1.00

0.94

1.02

0.99

1.33

1.13

0.83 – 1.06

0.91 – 1.13

0.98 – 0.99

1.25 – 1.41

0.94 – 1.37

0.00

1.00

0.00

0.00

0.00

0.98

1.30

1.14

0.98 – 0.99

1.24 – 1.37

1.04 – 1.26

0.00

0.00

0.00

1.14

1.25

1.25

1.25

1.08 – 1.21

1.18 – 1.33

1.18 – 1.33

1.18 – 1.33

0.00

0.00

0.00

0.00

1.15

1.24

1.32

1.38

1.01

1.08 – 1.22

1.16 – 1.32

1.20 – 1.45

1.19 – 1.60

1.01 – 1.02

0.00

0.00

0.00

0.00

0.00

*Mildly frail*

*Moderately frail*

*Severely frail*

Concurrent prescriptions

1.01

1.01 – 1.02

0.00

Usual provider of care index (ref: Quartile 4—Most

continuity)

[(Return to contents)](#br1)


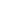

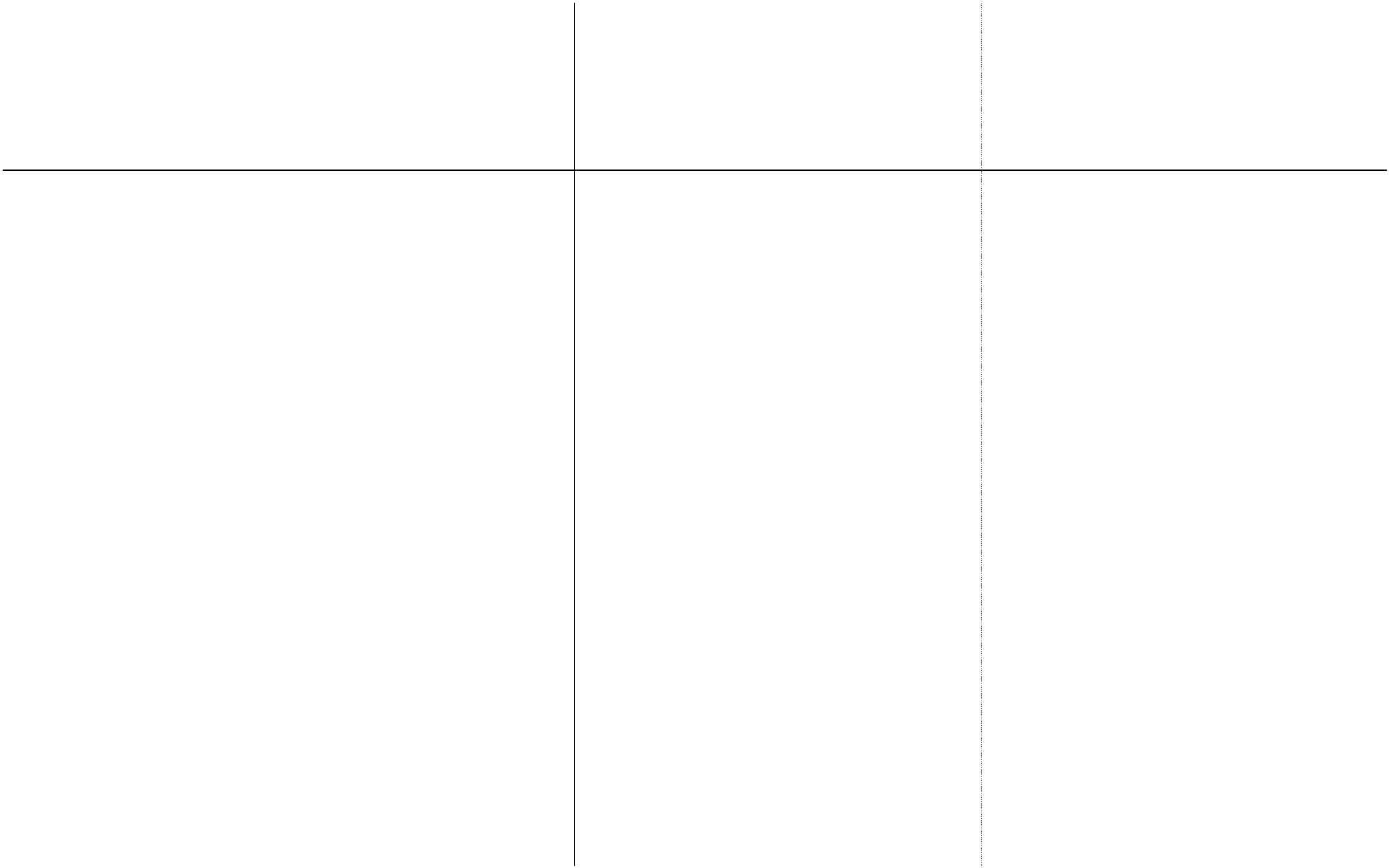


JA Villars et al. 15

**Primary outcome:**

**Secondary outcome:**

**Filled PPI prescription exceeding**

**the approved treatment duration**

**Filled PPI prescription exceeding**

**the approved treatment duration**

**accounting for gastroprotection**

**guidelines**

aHR

0.94

0.98

1.01

0.99

95% CI

0.89 – 1.00

0.93 – 1.04

0.95 – 1.07

0.99 – 1.00

p

0.04

0.59

0.82

0.00

aHR

0.95

0.97

1.01

1.00

1.05

95% CI

0.89 – 1.02 0.15

0.91 – 1.04

0.94 – 1.07

0.99 – 1.01

0.99 – 1.12

p

*Quartile 1—Least continuity*

*Quartile 2*

*Quartile 3*

VA priority group

Rural acute PUD diagnosing facility

**Time-varying factors**

Anticoagulants

Antiplatelets

Aspirin

H2-receptor antagonists

NSAIDs

0.00

1.00

0.00

0.00

1.06

1.00 – 1.13

0.05

1.33

1.20

1.25

1.08

1.26

1.20 – 1.48

1.08 – 1.32

1.17 – 1.34

0.77 – 1.52

1.19 – 1.34

0.00

0.00

0.00

1.00

0.00

1.24

1.16

1.22

1.10

1.27

1.10 – 1.41

0.93 – 1.44

1.12 – 1.33

0.69 – 1.74

1.12 – 1.45

0.00

0.00

0.00

1.00

0.00

[(Return to contents)](#br1)


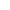

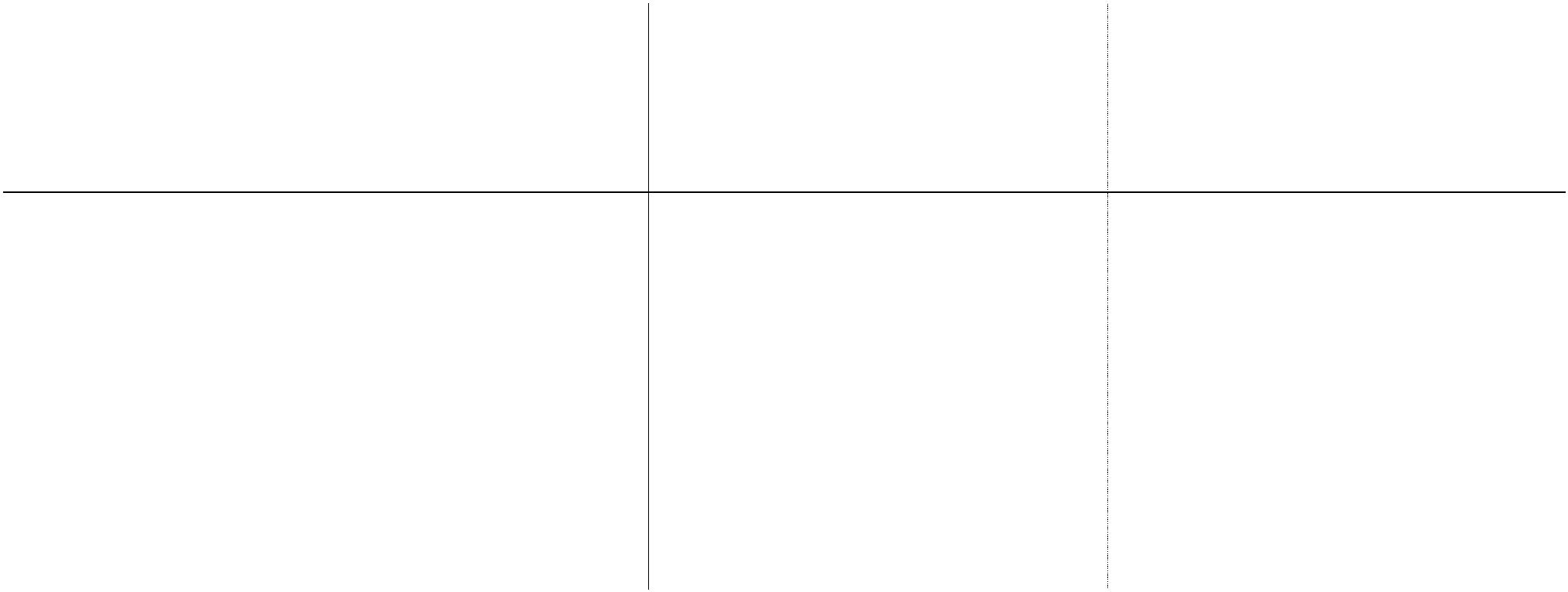


JA Villars et al. 16

**Supplemental table 6. Sensitivity analysis results for the primary outcome**

**No guideline-**

**Main analysis**

**discordant PPIs**

**60 days after**

**endoscopy**

**(primary**

**outcome)**

**5-year maximum**

**follow-up**

**Clopidogrel-PPI**

**warning**

**Modified VA**

**Frailty Index**

aHR

1.00

0.91

95% CI

1.00 –

1.01

0.83 –

0.99

aHR

1.00

–

95% CI

1.00 –

1.01

aHR

1.00

0.91

95% CI

1.00 –

1.01

0.83 –

0.99

aHR

1.00

–

95% CI

1.00 –

1.01

aHR

1.00

0.91

95% CI

1.00 –

1.01

0.83 –

0.99

Age

Female

–

–

Race (ref: White)

*Indigenous American or*

*Alaska Native*

0.84 –

1.24

0.74 –

1.26

0.78 –

0.88

0.95 –

1.34

0.93 –

1.10

0.98 –

0.99

0.70 –

1.12

0.85 –

1.45

0.79 –

0.90

0.88 –

1.33

0.87 –

1.04

0.97 –

0.98

0.75 –

1.16

0.80 –

1.33

0.81 –

0.91

0.91 –

1.34

0.88 –

1.04

0.97 –

0.98

0.68 –

1.13

0.79 –

1.44

0.81 –

0.92

0.93 –

1.48

0.84 –

1.06

0.97 –

0.98

0.75 –

1.16

0.79 –

1.33

0.81–

0.91

0.97 –

1.26

0.88 –

1.05

0.97 –

0.98

1.02

0.97

0.83

1.13

1.01

0.98

1.32

0.89

1.11

0.84

1.08

0.95

0.98

1.36

0.93

1.03

0.86

1.11

0.96

0.98

1.38

0.88

1.06

0.86

1.18

0.94

0.98

1.40

0.93

1.03

0.86

1.11

0.96

0.98

1.38

*Asian*

*Black*

*Native Hawaiian or Pacific*

*Islander*

*Declined/Unknown race*

Acute PUD diagnosis

calendar year

Inpatient acute PUD

diagnosis

1.25 –

1.39

1.28 –

1.44

1.31 –

1.46

1.31 –

1.49

1.31 –

1.45

VA frailty index (ref: Non-frail)

1.00 –

1.12

1.04 –

1.18

1.06 –

1.26

1.00 –

1.14

1.05 –

1.22

1.07 –

1.31

1.00 –

1.12

1.04 –

1.19

1.07 –

1.30

0.98 –

1.13

1.01 –

1.19

1.04 –

1.28

0.95 –

1.10

0.99 –

1.15

1.05 –

1.30

*Pre-frail*

1.06

1.11

1.15

1.08

1.07

1.14

1.18

1.18

1.06

1.12

1.18

1.14

1.05

1.09

1.15

1.09

1.02

1.07

1.16

1.08

*Mildly frail*

*Moderately frail*

*Severely frail*

0.94 –

1.23

1.02 –

1.37

0;99 –

1.32

0.93 –

1.27

0.95 –

1.24

[(Return to contents)](#br1)


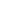

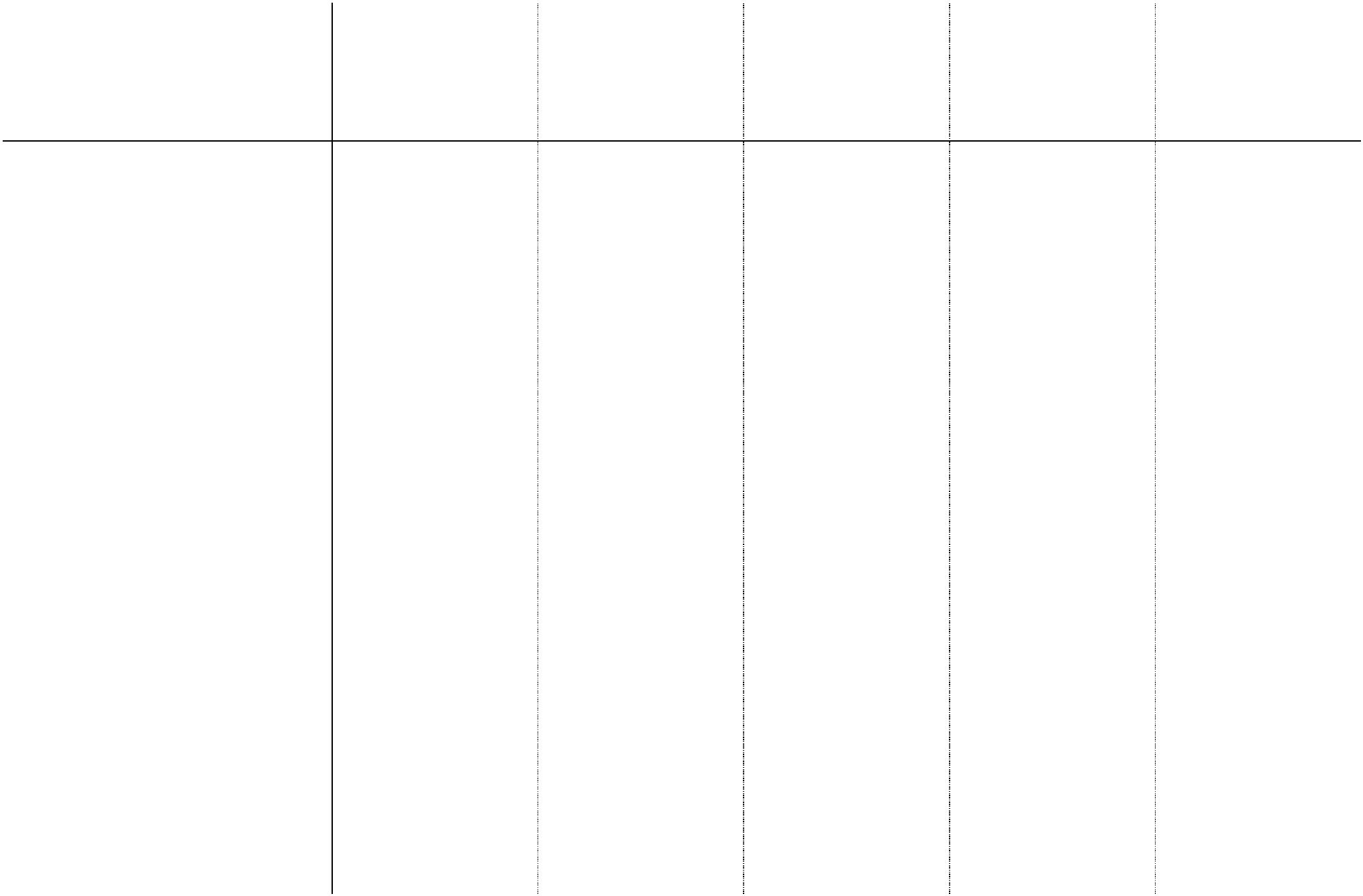


JA Villars et al. 17

0.98 –

1.00

1.01 –

1.01

1.01 –

1.15

0.98 –

1.00

1.01 –

1.01

1.00 –

1.13

Charlson-Deyo score

–

1.01

–

–

0.99

1.01

1.08

0.99

1.01

1.06

–

–

–

–

1.01 –

1.01

1.00 –

1.01

1.02 –

1.18

1.01 –

1.01

1.00 –

1.13

Concurrent prescriptions

1.01

1.10

1.01

1.07

Facility rurality

–

**Time-varying factors**

1.13 –

1.38

1.07 –

1.24

1.18 –

1.34

1.08 –

1.35

1.09 –

1.27

1.17 –

1.32

1.10 –

1.36

1.10 –

1.26

1.18 –

1.34

1.13 –

1.40

1.07 –

1.26

1.18 –

1.36

1.10 –

1.36

1.10 –

1.27

1.18 –

1.34

Anticoagulants

Aspirin

1.25

1.15

1.26

1.21

1.18

1.24

1.22

1.18

1.26

1.26

1.16

1.27

1.23

1.18

1.26

NSAIDs

[(Return to contents)](#br1)


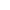

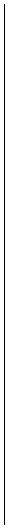

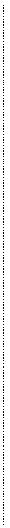

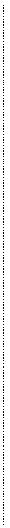

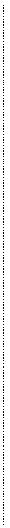

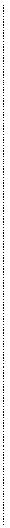


JA Villars et al. 18

**Supplemental table 7: ICD codes for chronic or unspecified PUD. Exclusions any time**

**before first acute PUD diagnosis.**

**Code**

**Description**

**Type**

Chronic or unspecified gastric ulcer with hemorrhage, without mention of

531.40 obstruction

ICD9

ICD9

531.41 Chronic or unspecified gastric ulcer with hemorrhage, with obstruction

Chronic or unspecified gastric ulcer with perforation, without mention of

531.60 obstruction

ICD9

ICD9

531.61 Chronic or unspecified gastric ulcer with perforation, with obstruction

Chronic or unspecified gastric ulcer with hemorrhage and perforation,

532.40 without mention of obstruction

Chronic or unspecified gastric ulcer with hemorrhage and perforation, with

532.41 obstruction

ICD9

ICD9

ICD9

ICD9

ICD9

Chronic gastric ulcer without mention of hemorrhage or perforation, without

532.60 mention of obstruction

Chronic gastric ulcer without mention of hemorrhage or perforation, with

532.61 obstruction

Gastric ulcer, unspecified as acute or chronic, without mention of

533.40 hemorrhage or perforation, with obstruction

Chronic or unspecified duodenal ulcer with hemorrhage, without mention of

533.41 obstruction

ICD9

ICD9

533.60 Chronic or unspecified duodenal ulcer with hemorrhage, with obstruction

Chronic or unspecified duodenal ulcer with perforation, without mention of

533.61 obstruction

ICD9

ICD9

K25.4 Chronic or unspecified duodenal ulcer with perforation, without obstruction

Chronic or unspecified duodenal ulcer with hemorrhage and perforation,

K25.6 without mention of obstruction

ICD9

ICD9

ICD9

ICD9

ICD9

ICD9

ICD9

ICD9

ICD9

ICD9

ICD9

Chronic or unspecified duodenal ulcer with hemorrhage and perforation,

K26.4 with obstruction

Chronic duodenal ulcer without mention of hemorrhage or perforation,

K26.6 without mention of obstruction

Chronic duodenal ulcer without mention of hemorrhage or perforation, with

K27.4 obstruction

Duodenal ulcer, unspecified as acute or chronic, without hemorrhage or

K27.6 perforation, without mention of obstruction

Chronic or unspecified peptic ulcer of unspecified site with hemorrhage,

533.40 without mention of obstruction

Chronic or unspecified peptic ulcer of unspecified site with hemorrhage,

533.41 without mention of obstruction

Chronic or unspecified peptic ulcer of unspecified site with hemorrhage,

533.60 with obstruction

Chronic or unspecified peptic ulcer of unspecified site with hemorrhage,

533.61 with obstruction

Chronic or unspecified peptic ulcer of unspecified site with perforation,

531.50 without mention of obstruction

Chronic or unspecified peptic ulcer of unspecified site with perforation, with

531.51 obstruction

[(Return to contents)](#br1)


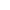

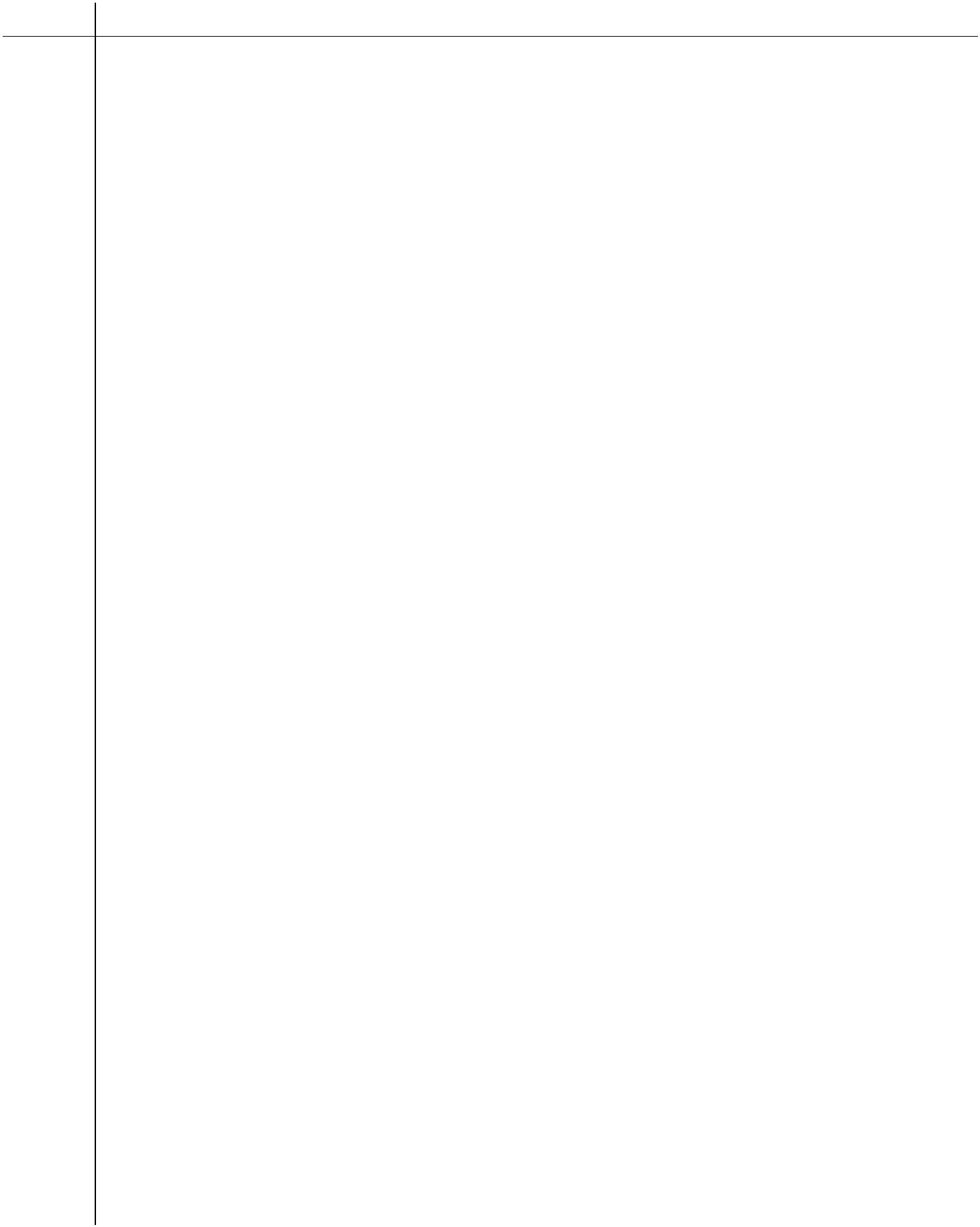


JA Villars et al. 19

**Code**

**Description**

Chronic or unspecified peptic ulcer of unspecified site with hemorrhage and

**Type**

531.70 perforation, without mention of obstruction

Chronic or unspecified peptic ulcer of unspecified site with hemorrhage and

531.71 perforation, without mention of obstruction

Chronic or unspecified peptic ulcer of unspecified site with hemorrhage and

531.91 perforation, with obstruction

Chronic or unspecified peptic ulcer of unspecified site with hemorrhage and

532.50 perforation, with obstruction

Chronic peptic ulcer of unspecified site without mention of hemorrhage or

532.51 perforation, without mention of obstruction

Chronic peptic ulcer of unspecified site without mention of hemorrhage or

532.70 perforation, with obstruction

ICD9

ICD9

ICD9

ICD9

ICD9

ICD9

Peptic ulcer of unspecified site, unspecified as acute or chronic, with

532.71 obstruction

ICD9

532.91 Chronic or unspecified gastric ulcer with hemorrhage

ICD10

ICD10

ICD10

ICD10

ICD10

ICD10

533.50 Chronic or unspecified gastric ulcer with perforation

533.51 Chronic or unspecified gastric ulcer with both hemorrhage & perforation

533.70 Chronic or unspecified gastric ulcer without perforation or perforation

533.71 Chronic or unspecified duodenal ulcer with hemorrhage

533.91 Chronic or unspecified duodenal ulcer with perforation

Chronic or unspecified duodenal ulcer with both hemorrhage and

K25.5 perforation

ICD10

ICD10

ICD10

ICD10

K25.7 Chronic or unspecified duodenal ulcer without perforation or perforation

K26.5 Chronic or unspecified peptic ulcer, site unspecified, with hemorrhage

K26.7 Chronic or unspecified peptic ulcer, site unspecified, with perforation

Chronic or unspecified peptic ulcer, site unspecified, with both hemorrhage

K27.5 and perforation

ICD10

ICD10

K27.7 Chronic peptic ulcer, site unspecified, without hemorrhage or perforation

Peptic ulcer, site unspecified, unspecified as acute or chronic, without

K27.9 hemorrhage or perforation

ICD10

[(Return to contents)](#br1)


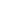

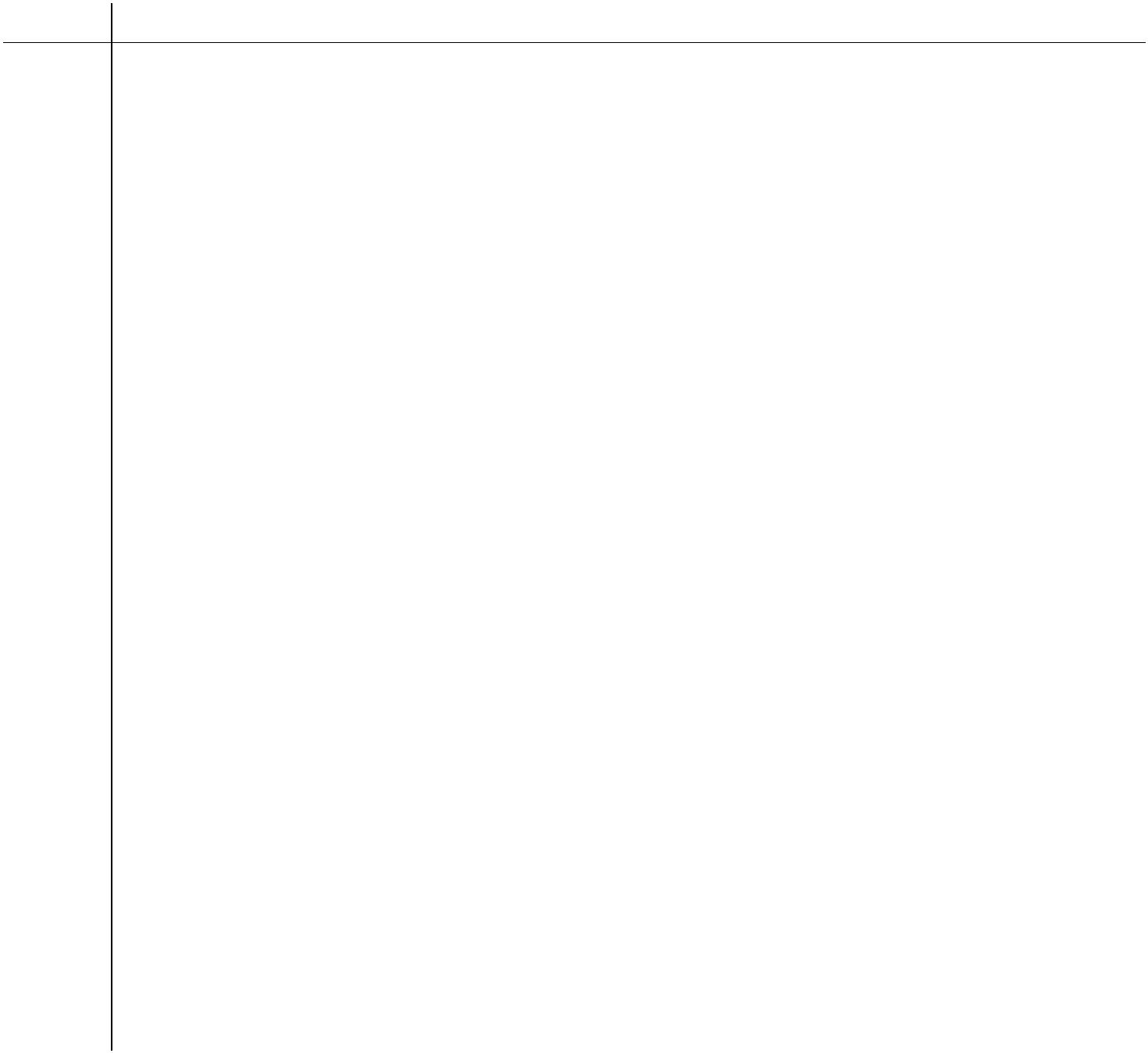


JA Villars et al. 20

**Supplemental table 8. ICD codes for indications for PPI use, upper gastrointestinal**

**malignancy, and altered upper gastrointestinal anatomy. Exclusions applied any time**

**before first PUD diagnosis.**

**Code**

150.0

150.1

150.2

150.3

150.4

150.5

150.8

150.9

151.0

151.1

151.2

151.3

151.4

151.5

151.6

151.8

151.9

152.0

152.8

152.9

251.5

530.10

530.11

530.12

530.13

530.19

530.20

530.21

530.81

530.82

534.00

534.01

534.10

534.11

534.20

534.21

**Description**

**Type**

ICD9

ICD9

ICD9

ICD9

ICD9

ICD9

ICD9

ICD9

ICD9

ICD9

ICD9

ICD9

ICD9

ICD9

ICD9

ICD9

ICD9

ICD9

ICD9

ICD9

ICD9

ICD9

ICD9

ICD9

ICD9

ICD9

ICD9

ICD9

ICD9

ICD9

ICD9

ICD9

ICD9

ICD9

ICD9

ICD9

Malignancy of Cervical esophagus

Malignancy of thoracic esophagus

Malignancy of abdominal esophagus

Malignancy of upper 1/3 of esophagus

Malignancy of middle 1/3 of esophagus

Malignancy of lower 1/3 of esophagus

Malignancy of other specified part of esophagus

Malignancy of esophagus, unspecified

Malignant neoplasm of cardia

Malignant neoplasm of pylorus

Malignant neoplasm of pyloric antrum

Malignant neoplasm of fundus of stomach

Malignant neoplasm of body of stomach

Malignant neoplasm of lesser curvature of stomach

Malignant neoplasm of greater curvature of stomach

Malignant neoplasm of other specified part of stomach

Malignant neoplasm of stomach, unspecified

Malignant neoplasm of duodenum

Malignant neoplasm of other specified sites of small intestine

Malignant neoplasm of small intestine, unspecified

Abnormality of secretion of gastrin (Zollinger-Ellison)

Esophagitis, unspecified

Reflux esophagitis

Acute esophagitis

Eosinophilic esophagitis

Other esophagitis

Ulcer of the esophagus w/o bleeding

Ulcer of the esophagus w/ bleeding

Esophageal reflux

Esophageal hemorrhage

Acute GJ ulcer w/ hemorrhage w/o obstruction

Acute GJ ulcer w/ perforation w/ obstruction

Acute GJ ulcer w/ perforation w/o obstruction

Acute GJ ulcer w/ perforation w/ obstruction

Acute GJ ulcer w/ perforation & hemorrhage w/o obstruction

Acute GJ ulcer w/ perforation & hemorrhage w/ obstruction

[(Return to contents)](#br1)


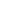

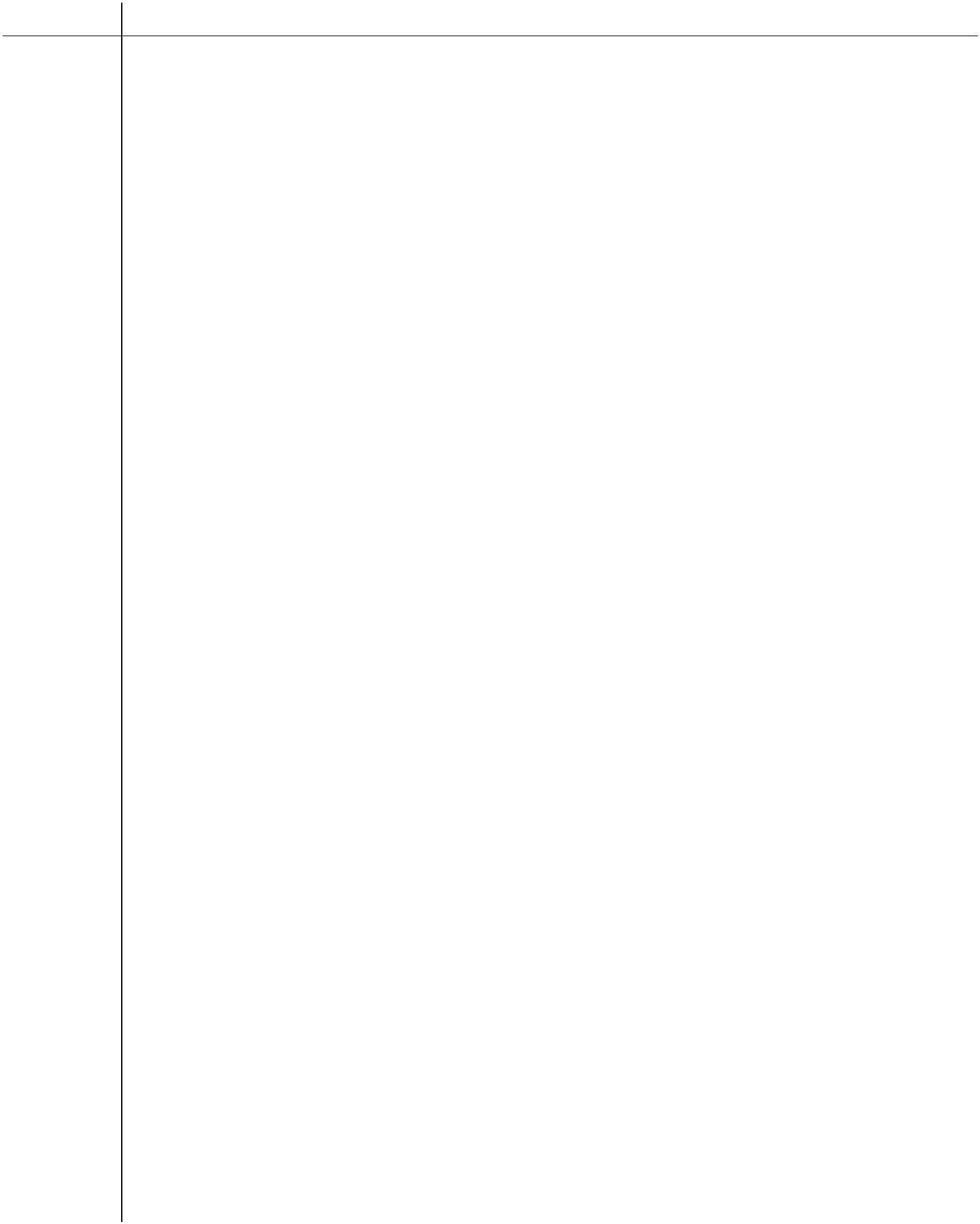


JA Villars et al. 21

**Code**

**Description**

**Type**

ICD9

ICD9

ICD9

ICD9

ICD9

ICD9

ICD9

ICD9

ICD9

ICD9

534.30

534.31

534.40

534.41

534.50

534.51

534.60

534.61

534.70

534.71

Acute GJ ulcer w/o perforation or hemorrhage w/o obstruction

Acute GJ ulcer w/o perforation or hemorrhage w/ obstruction

Chronic GJ ulcer w/ hemorrhage w/o obstruction

Chronic GJ ulcer w/ perforation w/ obstruction

Chronic GJ ulcer w/ perforation w/o obstruction

GJ ulcer w/ perforation w/ obstruction

Chronic GJ ulcer w/ perforation & hemorrhage w/o obstruction

Chronic GJ ulcer w/ perforation & hemorrhage w/ obstruction

Chronic GJ ulcer w/o perforation or hemorrhage w/o obstruction

Chronic GJ ulcer w/o perforation or hemorrhage w/ obstruction

Gastrojejunal ulcer, unspecified as acute or chronic, without mention of

hemorrhage or perforation, without mention of obstruction

Gastrojejunal ulcer, unspecified as acute or chronic, without mention of

hemorrhage or perforation, with obstruction

534.90

ICD9

534.91

C15.3

C15.4

C15.5

C15.8

C15.9

C16.0

C16.1

C16.2

C16.3

C16.4

C16.5

C16.6

C16.8

C16.9

C17.0

C17.8

C17.9

E16.4

K20.0

K20.8

K20.81

K20.9

K20.91

K21.00

K21.01

K21.9

ICD9

Intestinal bypass or anastomosis status

Acquired absence of organ, stomach

Bariatric surgery status

ICD9

ICD9

ICD9

Malignancy of upper 1/3 of esophagus

Malignancy of middle 1/3 of esophagus

Malignancy of lower 1/3 of esophagus

Malignancy of overlapping sites of esophagus

Malignancy of esophagus, unspecified

Malignant neoplasm of cardia

ICD10

ICD10

ICD10

ICD10

ICD10

ICD10

ICD10

ICD10

ICD10

ICD10

ICD10

ICD10

ICD10

ICD10

ICD10

ICD10

ICD10

ICD10

ICD10

ICD10

ICD10

ICD10

ICD10

Malignant neoplasm of fundus of stomach

Malignant neoplasm of body of stomach

Malignant neoplasm of pyloric antrum

Malignant neoplasm of pylorus

Malignant neoplasm of lesser curvature of stomach

Malignant neoplasm of greater curvature of stomach

Malignant neoplasm of overlapping sites of the stomach

Malignant neoplasm of stomach, unspecified

Malignant neoplasm of duodenum

Malignant neoplasm of overlapping sites of small intestine

Malignant neoplasm of small intestine, unspecified

Increased secretion of gastrin (Zollinger-Ellison)

Eosinophilic esophagitis

Other esophagitis w/o bleeding

Other esophagitis w/ bleeding

Esophagitis, unspecified, w/o bleeding

Esophagitis, unspecified, w/ bleeding

[(Return to contents)](#br1)


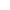

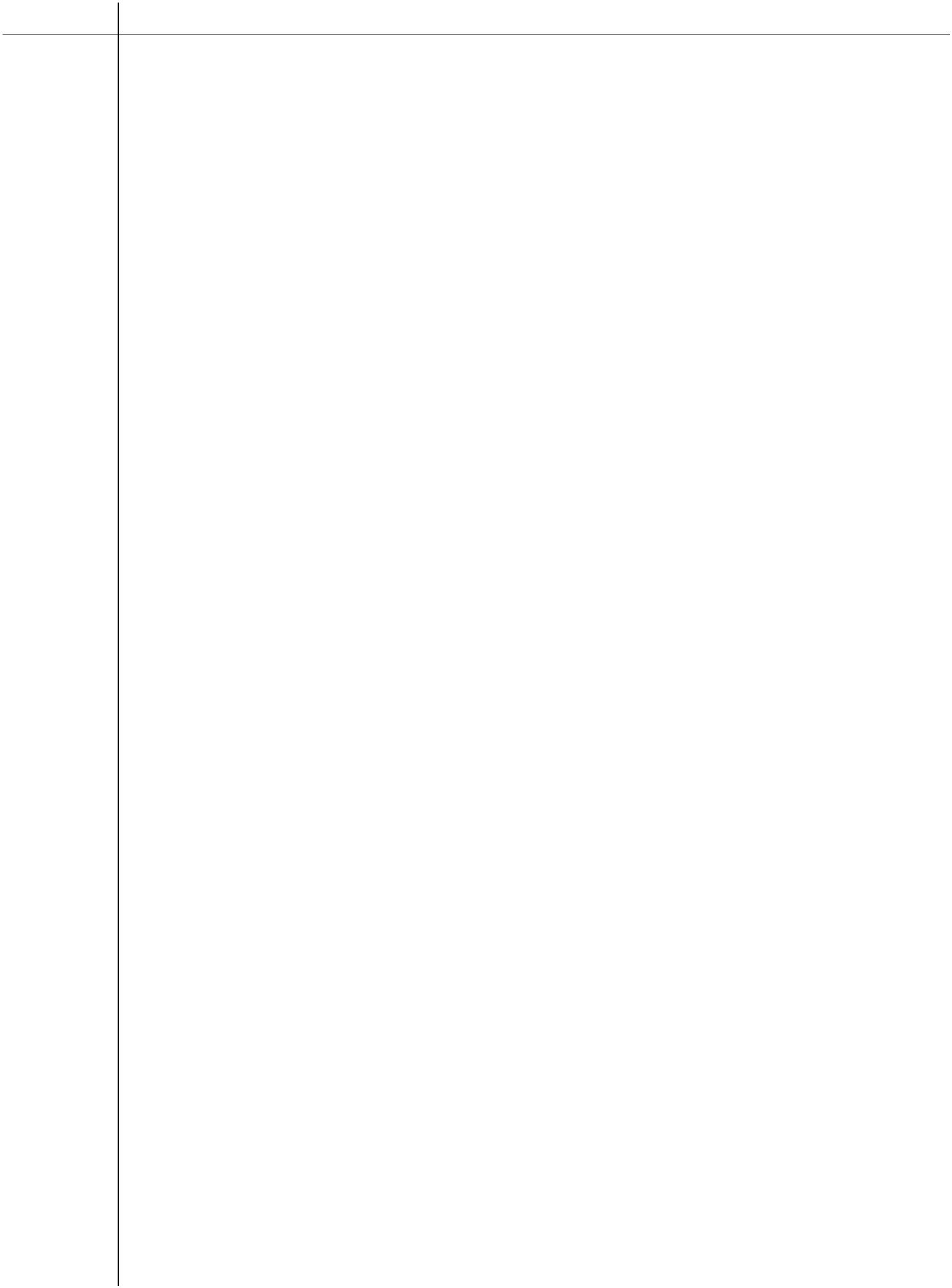


JA Villars et al. 22

**Code**

**Description**

**Type**

K22.10

K22.11

K22.6

Gastro-esophageal reflux disease with esophagitis w/ bleeding

Gastro-esophageal reflux disease with esophagitis w/o bleeding

Gastro-esophageal reflux disease without esophagitis

Ulcer of the esophagus w/o bleeding

ICD10

ICD10

ICD10

ICD10

ICD10

ICD10

ICD10

ICD10

ICD10

ICD10

ICD10

ICD10

ICD10

ICD10

ICD10

ICD10

ICD10

ICD10

K22.70

K22.710

K22.711

K22.719

K28.0

Ulcer of the esophagus w/ bleeding

Gastro-esophageal laceration-hemorrhage syndrome

Barrets esophagus w/o dyplasia

Barrets esophagus w/ low grade dyplasia

Barrets esophagus w/ high grade dyplasia

Barrets esophagus w/ unspecified dyplasia

Acute GJ ulcer w/ hemorrhage

K28.1

K28.2

K28.3

K28.4

Acute GJ ulcer w/ perforation

K28.5

Acute GJ ulcer w/ perforation & hemorrhage

Acute GJ ulcer w/o perforation or hemorrhage

Chronic GJ ulcer w/ hemorrhage

K28.6

K28.7

K28.9

Chronic GJ ulcer w/ perforation

V45.3

Chronic GJ ulcer w/ perforation & hemorrhage

V45.75

Chronic GJ ulcer w/o perforation or hemorrhage

GJ ulcer, unspecified as acute or chronic, without hemorrhage or

perforation

V45.86

Z90.3

Z98.0

Z98.4

ICD10

ICD10

ICD10

ICD10

Acquired absence of stomach (part of)

Intestinal bypass and anastomosis status

Bariatric surgery status

[(Return to contents)](#br1)


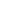

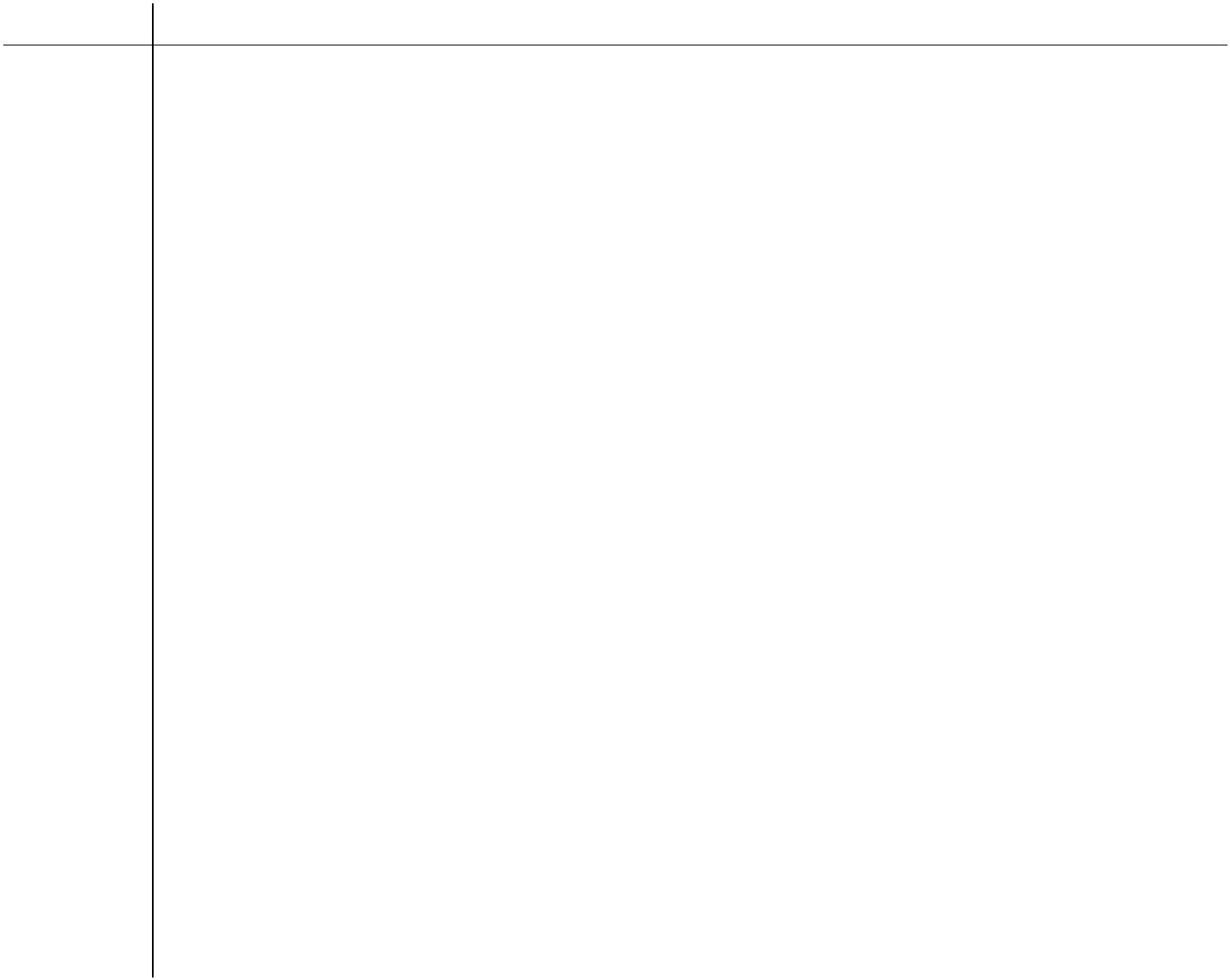


JA Villars et al. 23

**Supplemental table 9. ICD codes for non-PUD causes of GI bleeding. Exclusions applied**

**365 – 14 days before the first acute PUD diagnosis.**

**Code**

456.0

456.1

578.0

578.1

578.9

456.20

**Description**

**Type**

ICD9

ICD9

ICD9

ICD9

ICD9

ICD9

Esophageal varices with bleeding

Esophageal varices without mention of bleeding

Hematemesis

Blood in stool

Hemorrhage of gastrointestinal tract, unspecified

Esophageal varices in diseases classified elsewhere, with bleeding

Esophageal varices in diseases classified elsewhere, without mention

of bleeding

456.21

569.84

569.85

569.86

I85.00

I85.01

I85.10

I85.11

K31.811

K31.819

K31.82

K92.0

ICD9

Angiodysplasia of intestine w/o hemorrhage

Angiodysplasia of intestine w/ bleeding

Dieulafoy lesion (hemorrhagic) of intestines

Esophageal varices without bleeding

Esophageal varices with bleeding

ICD9

ICD9

ICD9

ICD10

ICD10

ICD10

ICD10

ICD10

ICD10

ICD10

ICD10

ICD10

ICD10

Secondary esophageal varices without bleeding

Secondary esophageal varices with bleeding

Angiodysplasia of stomach & duodenum w/ bleeding

Angiodysplasia of stomach & duodenum w/o bleeding

Dieulafoy lesion (hemorrhagic) of stomach & duodenum

Hematemesis

K92.1

Melena

K92.2

Gastrointestinal hemorrhage, unspecified

[(Return to contents)](#br1)


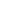

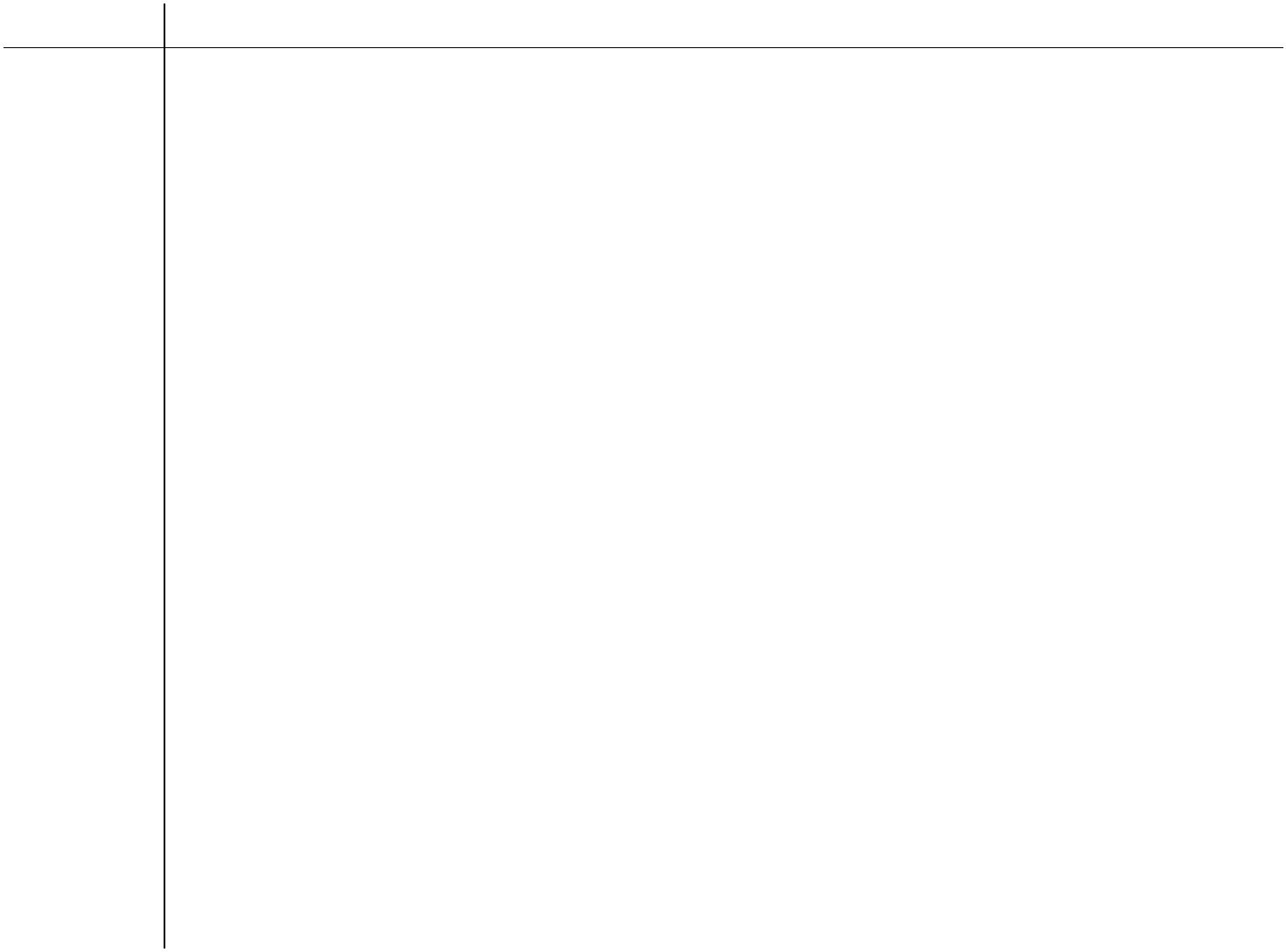


JA Villars et al. 24

**Supplemental table 10. Current Procedural Terminology-4 codes for upper endoscopy**

**Code**

**Description**

43233 Egd balloon dil esoph 30 mm

43235 Egd diagnostic brush wash

43236 Uppr gi scope w/submuc inj

43237 Endoscopic us exam esoph

43238 Egd us fine needle bx/aspir

43239 Egd biopsy single/multiple

43240 Egd w/transmural drain cyst

43241 Egd tube/cath insertion

43242 Egd us fine needle bx/aspir

43244 Egd varices ligation

43245 Egd dilate stricture

43246 Egd place gastrostomy tube

43247 Egd remove foreign body

43248 Egd guide wire insertion

43249 Esoph egd dilation <30 mm

43250 Egd cautery tumor polyp

43251 Egd remove lesion snare

43252 Egd optical endomicroscopy

43253 Egd us transmural injxn/mark

43254 Egd endo mucosal resection

43255 Egd control bleeding any

43256 Egd us exam duodenum/jejunum

43257 Treatment of GERD w/ thermal energy

Egd with ablation of tumor(s), polyp(s) or other lesion(s) not amenable to removal by hot

43258 biopsy forceps, bipolar cautery or snare technique (deleted 01/01/2014)

43259 Additional EGDUS code

43266 Egd endoscopic stent place

43270 Egd lesion ablation

44360 Push enteroscopy - Endoscopic exam of duodenum/jejunum + brushings/washings

44361 Push enteroscopy - Endoscopic exam of duodenum/jejunum + biopsies

Push enteroscopy - Endoscopic exam of duodenum/jejunum + Removal of swallowed

44363 objects

Push enteroscopy - Endoscopic exam of duodenum/jejunum + polyp/tumor/lesion

44364 removal via snare

Push enteroscopy - Endoscopic exam of duodenum/jejunum + polyp/tumor/lesion

44365 removal via hot forceps/cautery

Push enteroscopy - Endoscopic exam of duodenum/jejunum + control of bleeding by

44366 any method

Push enteroscopy - Endoscopic exam of duodenum/jejunum + polyp/tumor/lesion

44369 removal via laser/RF

[(Return to contents)](#br1)


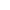

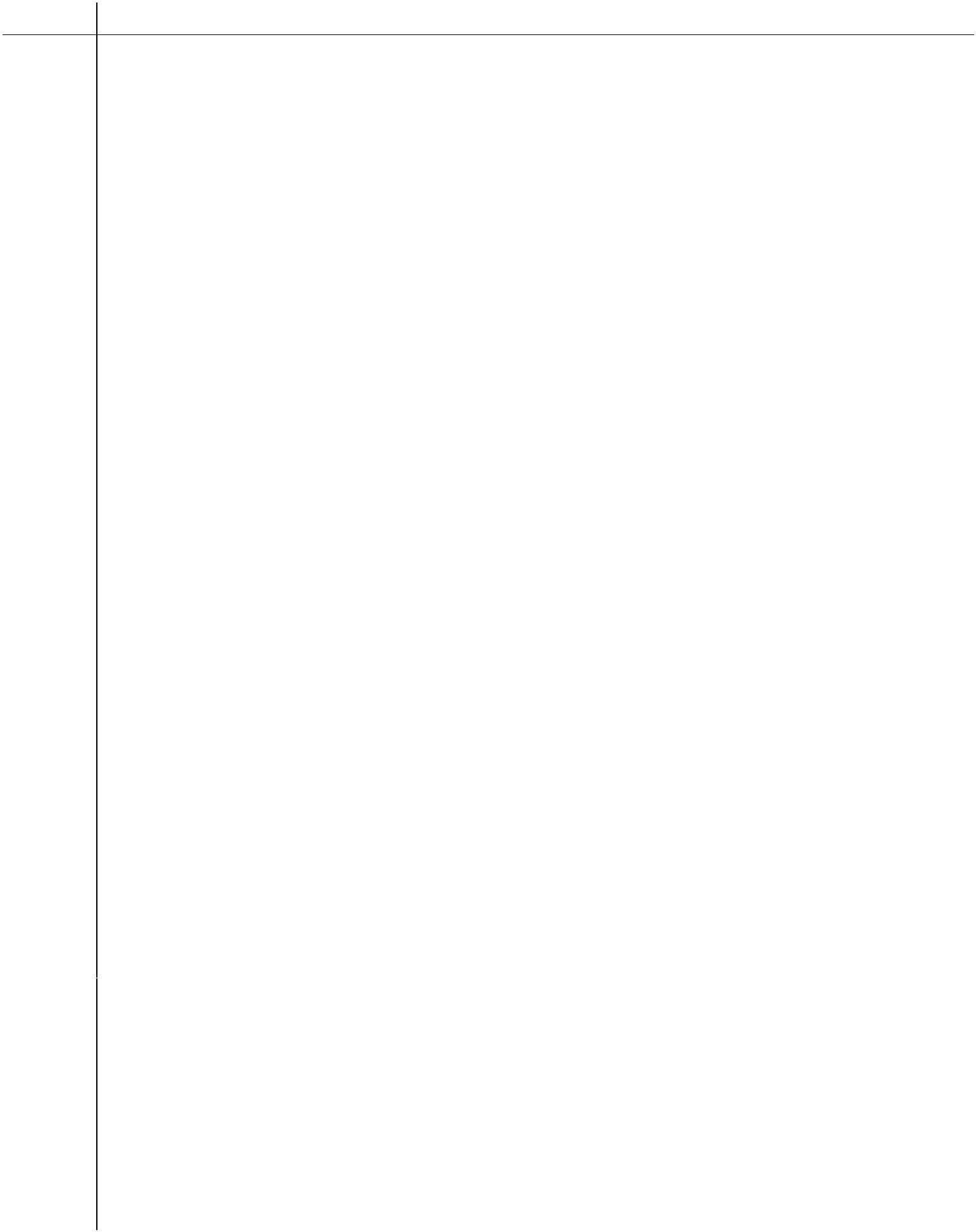


JA Villars et al. 25

**Code**

**Description**

44370 Push enteroscopy - Endoscopic exam of duodenum/jejunum + dilation

44372 Push enteroscopy - Endoscopic exam of duodenum/jejunum + J tube placement

44373 Push enteroscopy - Endoscopic exam of duodenum/jejunum + convert GT to JT

44376 Endoscopic exam of Entire UGIT + brushings/washings

44377 Endoscopic exam of Entire UGIT + biopsies

44378 Endoscopic exam of Entire UGIT + control of bleeding by any method

44379 Endoscopic exam of Entire UGIT + dilation & stent placement

[(Return to contents)](#br1)


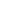

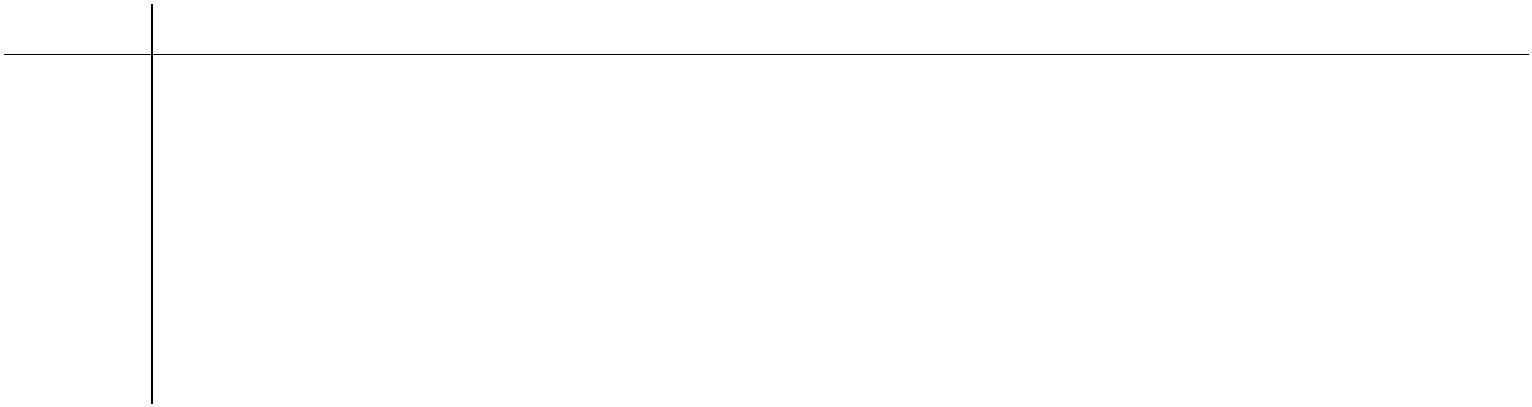


JA Villars et al. 26

**Supplemental figure 1. Conceptual model for relationship of assessed healthcare factors with filled PPI prescriptions**

**exceeding the approved treatment duration**

Notes: Solid boxes indicate measured healthcare factors. Dashed boxes indicate unobserved constructs. Arrows indicate

association, but do not indicate whether the association is positive or inverse.

[(Return to contents)](#br1)


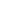

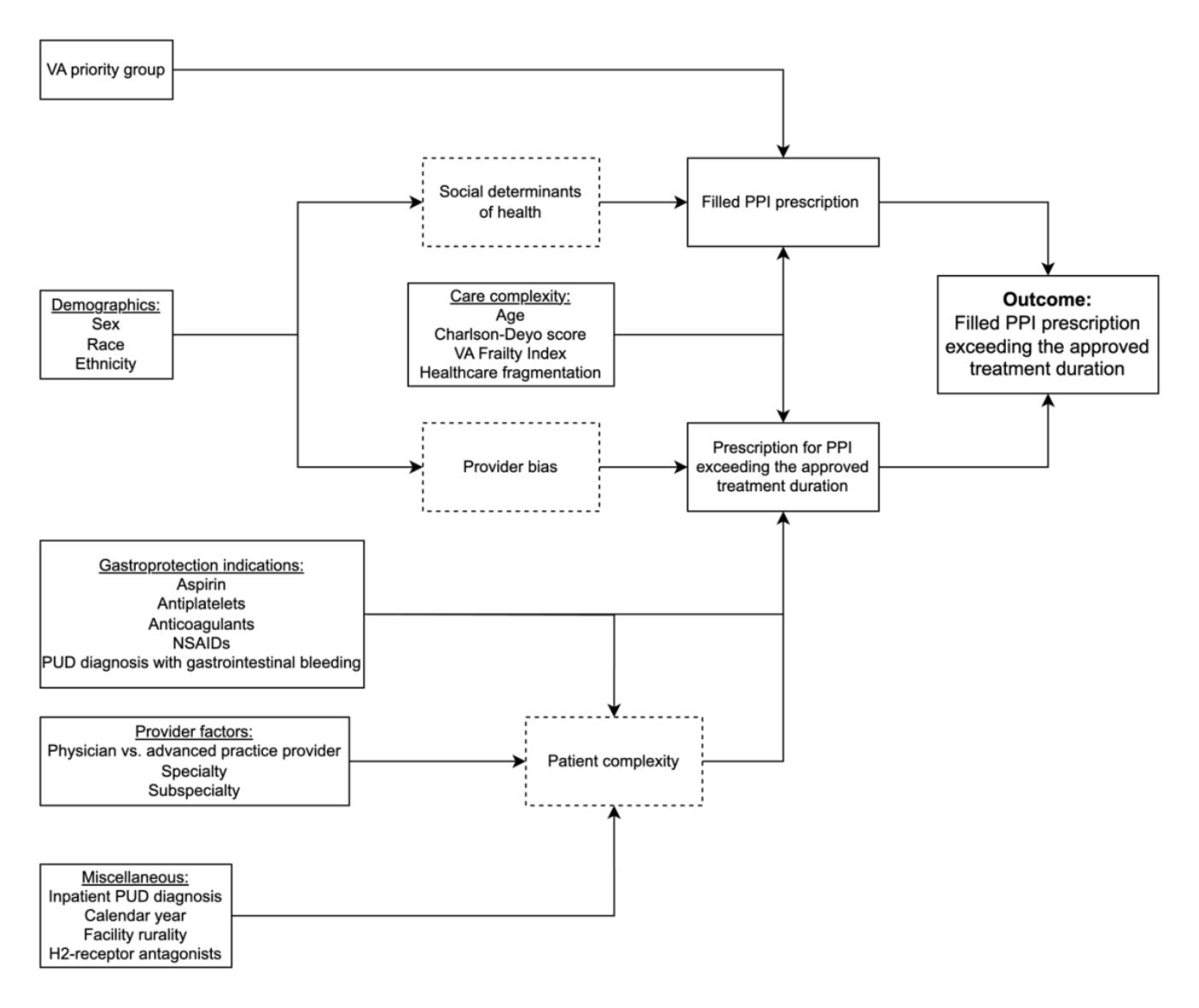


JA Villars et al. 27

**Supplemental figure 2. Assessment intervals for cohort eligibility, outcome, and factor criteria**

[(Return to contents)](#br1)


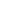


JA Villars et al. 28

[(Return to contents)](#br1)


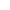

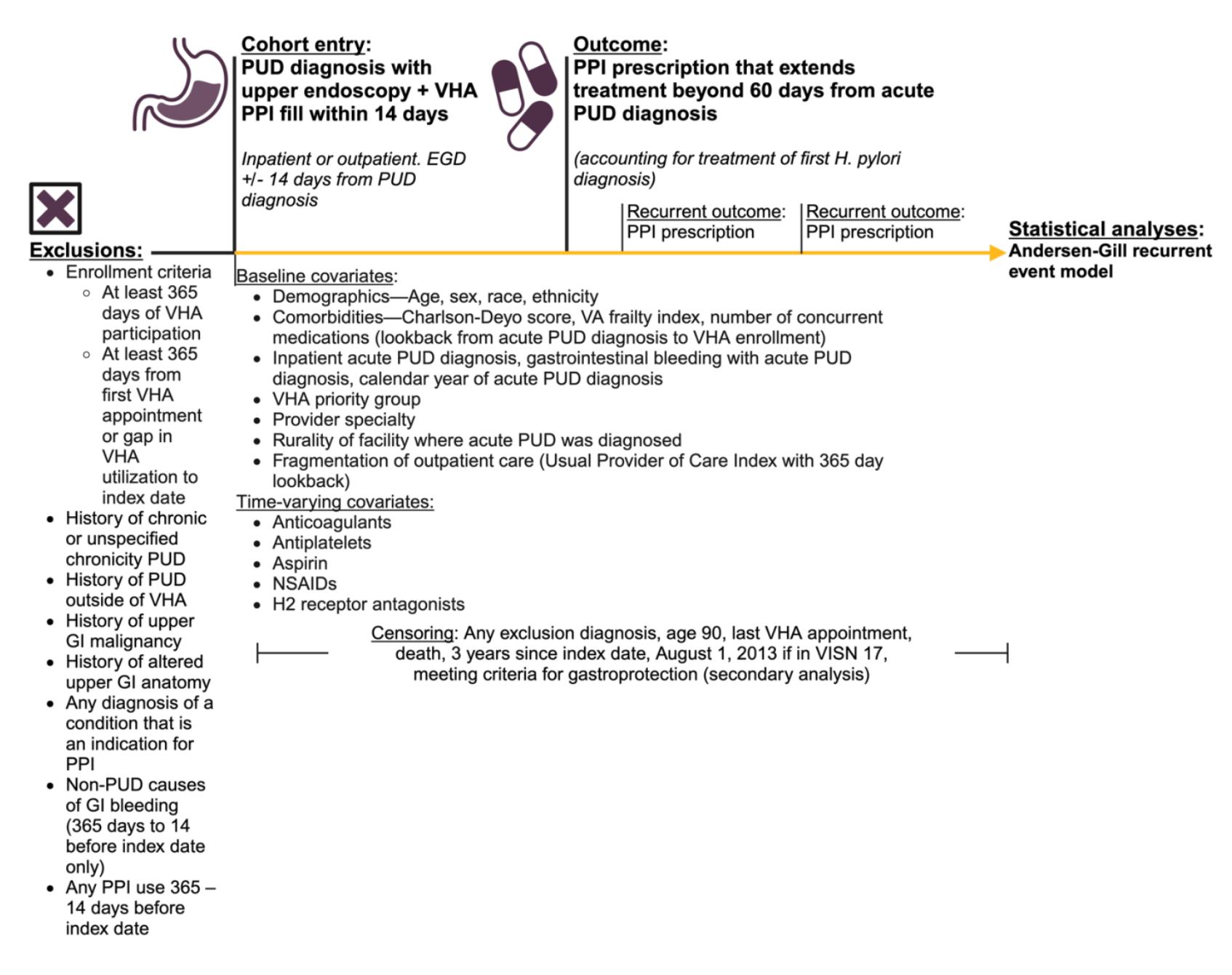


JA Villars et al. 29

**Supplemental figure 3. Adjusted population-attributable fraction for selected factors**

**positively associated with PPI prescriptions exceeding the approved treatment duration**

**(secondary outcome).**

Note: The population attributable fraction represents the proportion of PPI prescriptions that

would be eliminated if the factor was not present in the population.

[(Return to contents)](#br1)


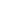

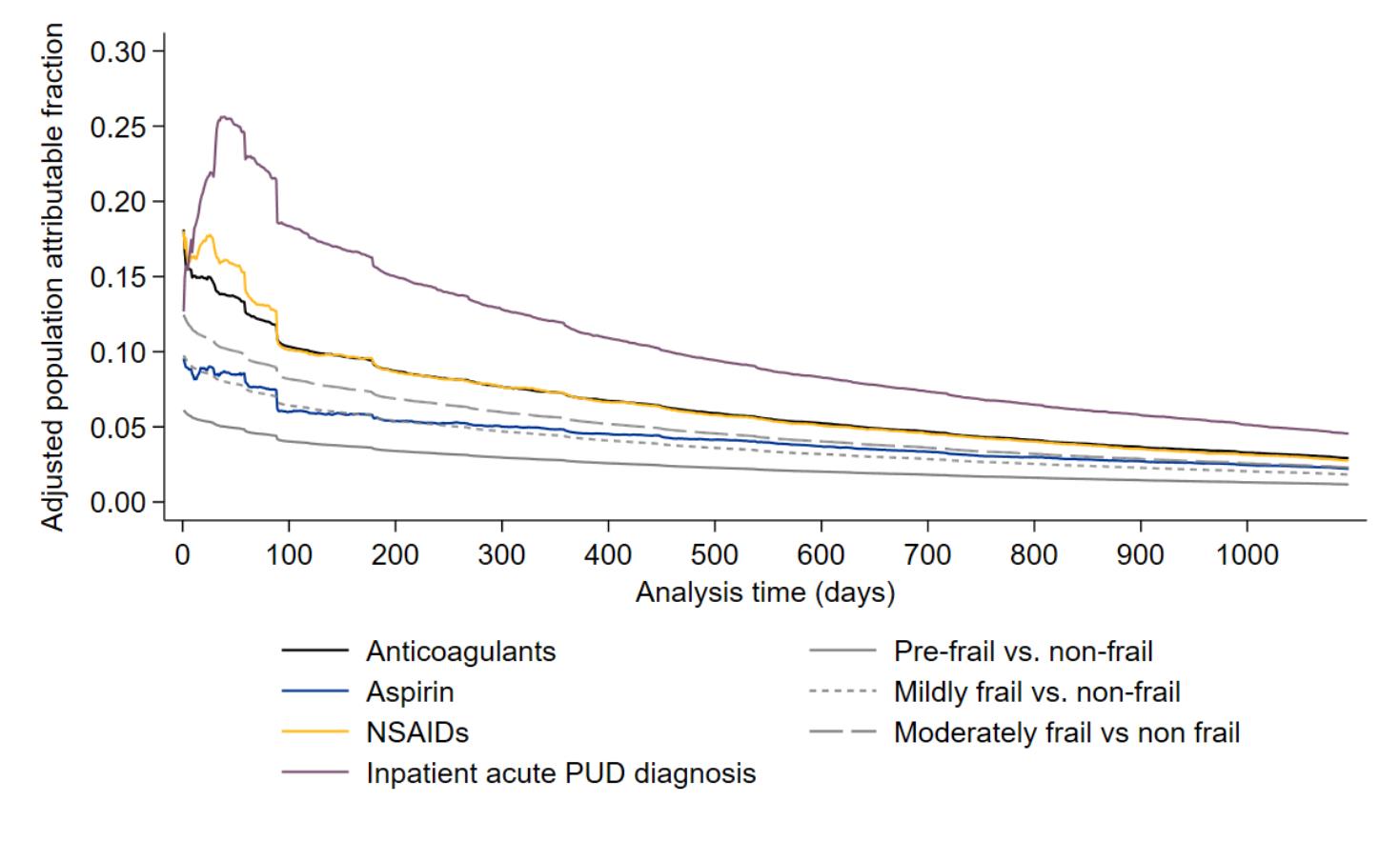

Supplement: Supplementary file 1 — Data S1. Supporting Information. [file PDS-34-e70152-s002.docx]
