# Supplementary material for: Proton Pump Inhibitor Use Exceeding the U.S. Food and Drug Administration Approved Treatment Duration for Patients With Peptic Ulcer Disease: A Retrospective Cohort Study
Source: Pharmacoepidemiol Drug Saf. 2025 Apr 29;34(5):e70152. doi: 10.1002/pds.70152 (PMC12038380; doi:10.1002/pds.70152)

Proton Pump Inhibitor Use Exceeding the U.S. Food and Drug Administration Approved Treatment Duration for Patients

with Peptic Ulcer Disease: A Retrospective Cohort Study

STROBE Statement—checklist of items that should be included in reports of observational studies

**Item**

**No.**

**Page** /section

**Recommendation**

(*a*) Indicate the study’s design with a commonly used term in the title or the abstract

(*b*) Provide in the abstract an informative and balanced summary of what was done and what was

found

1

**Title and abstract**

1

6

**Introduction**

Background/rationale

Objectives

2

3

Explain the scientific background and rationale for the investigation being reported

State specific objectives, including any prespecified hypotheses

8 - 9

9

**Methods**

Study design

Setting

4

5

Present key elements of study design early in the paper

10 - 12

Describe the setting, locations, and relevant dates, including periods of recruitment, exposure,

follow-up, and data collection

9

Participants

6

(*a*) *Cohort study*—Give the eligibility criteria, and the sources and methods of selection of

participants. Describe methods of follow-up

10

*Case-control study*—Give the eligibility criteria, and the sources and methods of case

ascertainment and control selection. Give the rationale for the choice of cases and controls

*Cross-sectional study*—Give the eligibility criteria, and the sources and methods of selection of

participants

(*b*) *Cohort study*—For matched studies, give matching criteria and number of exposed and

unexposed

N/A

*Case-control study*—For matched studies, give matching criteria and the number of controls per

case

Variables

7

Clearly define all outcomes, exposures, predictors, potential confounders, and effect modifiers.

Give diagnostic criteria, if applicable

10 - 12

Data sources/

measurement

Bias

8*

For each variable of interest, give sources of data and details of methods of assessment

(measurement). Describe comparability of assessment methods if there is more than one group

Describe any efforts to address potential sources of bias

9

11 - 13

9

Study size

10

Explain how the study size was arrived at

15 - 16

Continued on next page

1


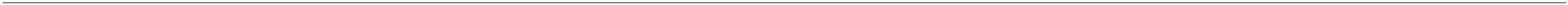

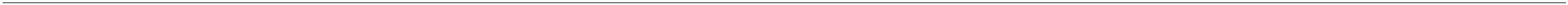

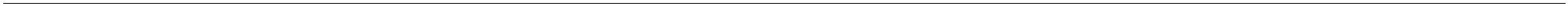

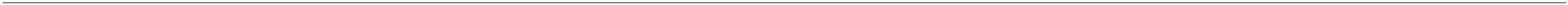

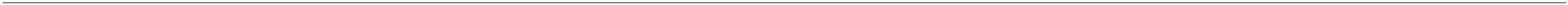

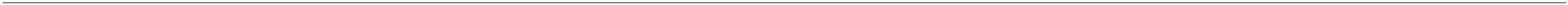

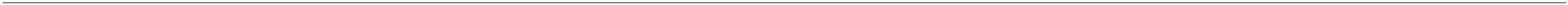

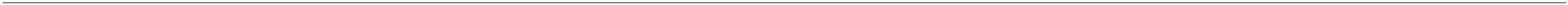

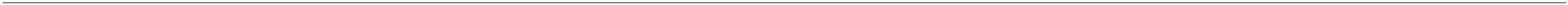

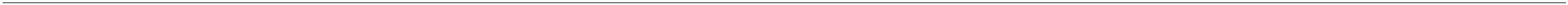

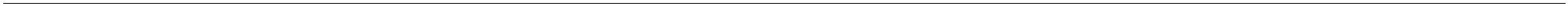

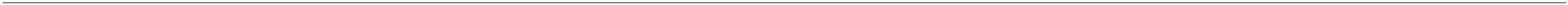

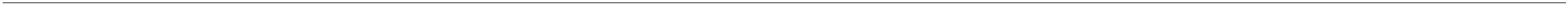

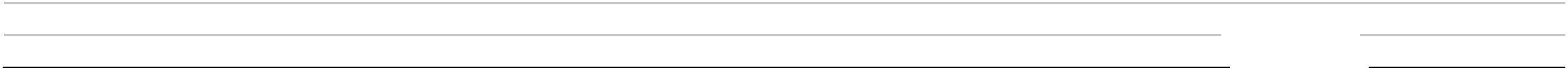

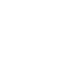


Quantitative

variables

11

12

Explain how quantitative variables were handled in the analyses. If applicable, describe which

groupings were chosen and why

12 - 14

13 - 14

14 - 15

Statistical

methods

(*a*) Describe all statistical methods, including those used to control for confounding

(*b*) Describe any methods used to examine subgroups and interactions

(*c*) Explain how missing data were addressed

SMethods

(*d*) *Cohort study*—If applicable, explain how loss to follow-up was addressed

*Case-control study*—If applicable, explain how matching of cases and controls was addressed

*Cross-sectional study*—If applicable, describe analytical methods taking account of sampling

strategy

13

(*e*) Describe any sensitivity analyses

13

**Results**

Participants

13*

14*

(a) Report numbers of individuals at each stage of study—eg numbers potentially eligible, examined

for eligibility, confirmed eligible, included in the study, completing follow-up, and analysed

(b) Give reasons for non-participation at each stage

15 F2

F2

(c) Consider use of a flow diagram

F2

Descriptive data

(a) Give characteristics of study participants (eg demographic, clinical, social) and information on

exposures and potential confounders

T1

(b) Indicate number of participants with missing data for each variable of interest

(c) *Cohort study*—Summarise follow-up time (eg, average and total amount)

*Cohort study*—Report numbers of outcome events or summary measures over time

*Case-control study—*Report numbers in each exposure category, or summary measures of exposure

*Cross-sectional study—*Report numbers of outcome events or summary measures

(*a*) Give unadjusted estimates and, if applicable, confounder-adjusted estimates and their precision

(eg, 95% confidence interval). Make clear which confounders were adjusted for and why they were

included

N/A

ST3

15

Outcome data

Main results

15*

16

ST5

(*b*) Report category boundaries when continuous variables were categorized

(*c*) If relevant, consider translating estimates of relative risk into absolute risk for a meaningful time

period

N/A

16

Continued on next page

2


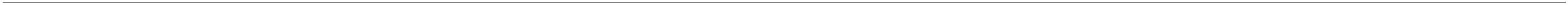

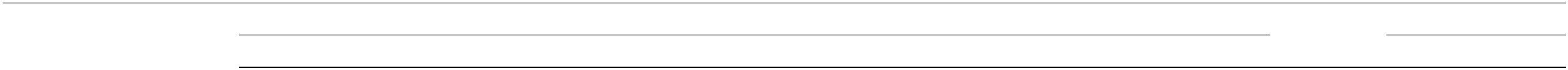

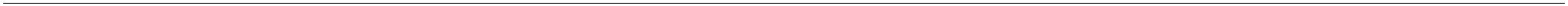

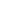

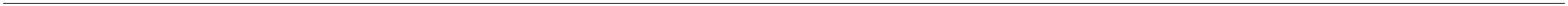

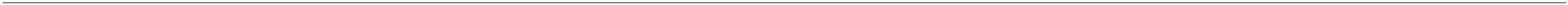

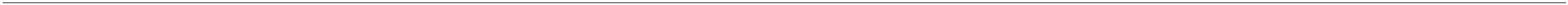

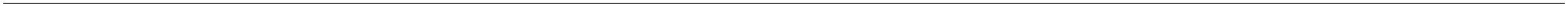

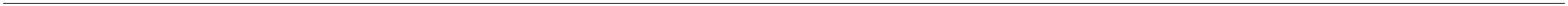

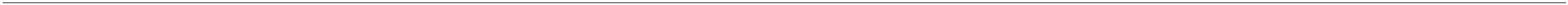

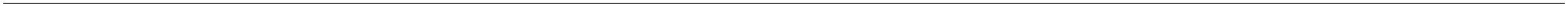

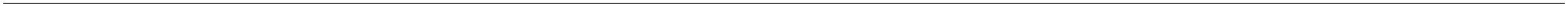

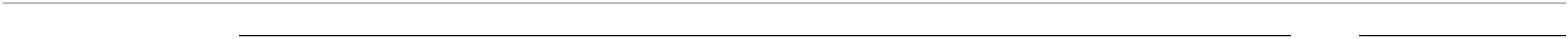

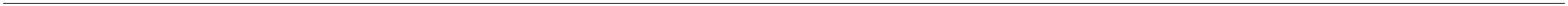

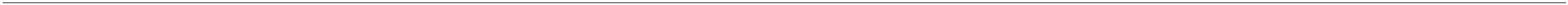

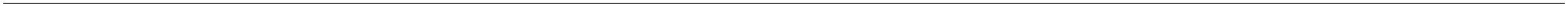

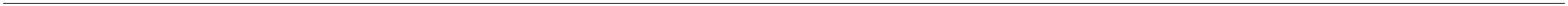

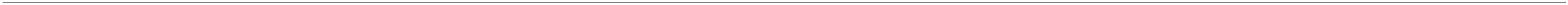

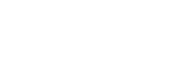

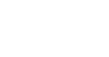

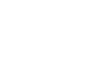


17 ST6

Other analyses

17 Report other analyses done—eg analyses of subgroups and interactions, and sensitivity analyses

**Discussion**

Key results

Limitations

18 Summarise key results with reference to study objectives

17 - 18

19 Discuss limitations of the study, taking into account sources of potential bias or imprecision. Discuss

both direction and magnitude of any potential bias

19

Interpretation

20 Give a cautious overall interpretation of results considering objectives, limitations, multiplicity of

analyses, results from similar studies, and other relevant evidence

20

19

Generalisability 21 Discuss the generalisability (external validity) of the study results

**Other information**

Funding

22 Give the source of funding and the role of the funders for the present study and, if applicable, for the

original study on which the present article is based

2

*Give information separately for cases and controls in case-control studies and, if applicable, for exposed and unexposed groups in cohort and cross-sectional studies.

**Note:** An Explanation and Elaboration article discusses each checklist item and gives methodological background and published examples of transparent reporting. The STROBE

checklist is best used in conjunction with this article (freely available on the Web sites of PLoS Medicine at http://www.plosmedicine.org/, Annals of Internal Medicine at

http://www.annals.org/, and Epidemiology at http://www.epidem.com/). Information on the STROBE Initiative is available at www.strobe-statement.org.

3


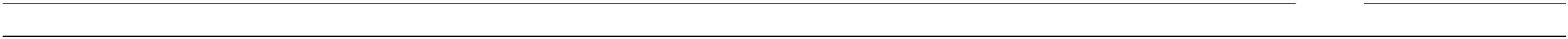

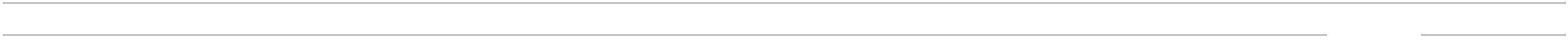

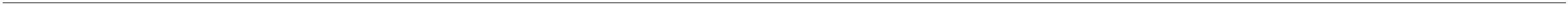

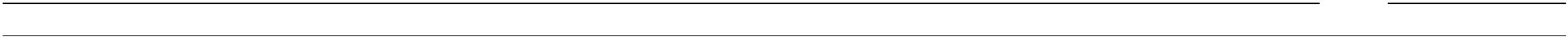

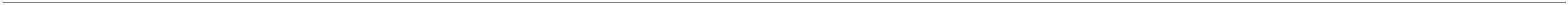

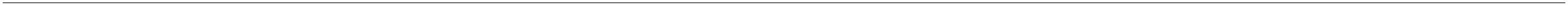

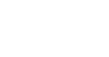

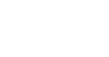

Supplement: Supplementary file 2 — Data S2. Supporting Information. [file PDS-34-e70152-s001.docx]
